# A Thermoreversible Gelation Pathway to Plant Cuticle‐Inspired Biopolymer Aerogel Monoliths Based on Network‐Spherulitic Crystal Assemblies

**DOI:** 10.1002/adma.75136

**Published:** 2026-09-29

**Authors:** Anthony V. Tuccitto, Zeineb Ben Rejeb, Rafaela Aguiar, Nello D. Sansone, Sumaiya Farzana, Isaac Ali, Jun Uk Lee, Daniel J. Davidson, Ailsa K. Edward, Chul B. Park, Patrick C. Lee

**Affiliations:** ^1^ Multifunctional Composites Manufacturing Laboratory (MCML) Department of Mechanical and Industrial Engineering University of Toronto Toronto Ontario Canada; ^2^ Microcellular Plastics Manufacturing Laboratory (MPML) Department of Mechanical and Industrial Engineering University of Toronto Toronto Ontario Canada; ^3^ Department of Chemical Engineering and Applied Chemistry University of Toronto Toronto Ontario Canada; ^4^ Department of Chemistry Lash Miller Chemical Laboratories University of Toronto Toronto Ontario Canada

**Keywords:** Hierarchical structure, Macro‐/mesoporous, Polycaprolactone, Poly(lactide), Superhydrophobic, Synchrotron SAXS, Thermogels

## Abstract

Plant‐based and compostable biopolymers are vital in global shifts toward green materials. However, their adoption in aerogel fabrication is limited by material and structure‐performance deficiencies (e.g., hydrophilicity, low surface area, brittleness, limited tactile tailorability). Nevertheless, stereocomplexation, or co‐crystallization between polymeric enantiomers, offers a pathway toward overcoming such limitations. Herein, stereocomplexation is used to fabricate poly(lactide) (PLA)‐based aerogels, with stereocomplex crystals (SCs) assembling into a highly ordered morphology/topology within aerogel precursors. To tailor aerogel bulk properties, a blending approach is implemented during sol–gel processing, leading to sequential crystallization of sol–gel system constituents. This pathway yields hierarchically macro‐/meso‐porous aerogels (*S*_BET_ up to ∼186 m^2^·g^−1^), offering a variety of superstructures that resemble cuticular plant tissue. These aerogels resist brittle failure under compression (70% strain) and withstand cyclic loading (100 cycles, 20% strain) without significant densification. Such aerogels also exhibit functional properties including superhydrophobicity/superoleophilicity and are well‐suited for continuous oil–water separation. Additionally, fantastic geometric‐thermal stability, insulation/heat dissipation ability (*k*_Cond_ ∼36 mW·m^−1^·K^−1^), and phase‐change insulation characteristics are achieved. These functionalities arise from multi‐scale structural features and demonstrate how coupling stereocomplexation and sequential crystallization offers pathways to construct intricate superstructure morphologies/topologies, and overcome material/structure‐performance limitations in green aerogels.

## Graphical abstract

To address functionality limitations in plant‐based and industrially compostable polymer aerogels, this work introduces bio‐inspired aerogels with broadened functionality. Inspired by functional plant tissues, lightweight biopolymer aerogels based on exclusively stereocomplex crystals are fabricated, featuring hierarchically macro‐/meso‐porous crystalline structures, similar to structures found in plant cuticles. The unique structures developed herein offer more user‐friendly biopolymer aerogels, with tailorable micro‐/nano‐structures and thus, bulk properties.

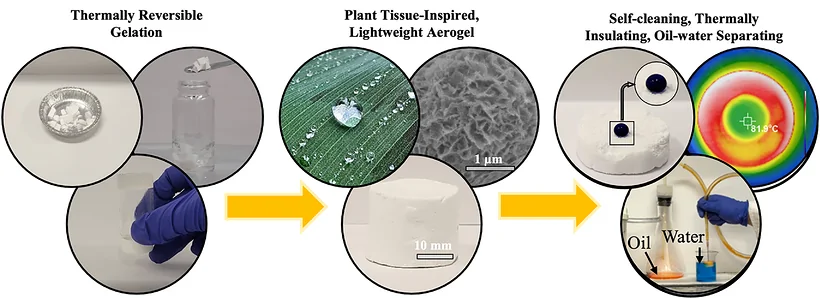

## 1 Introduction

Silica, carbon, and graphene are traditional materials used as precursors for aerogels, offering extremely low densities (typically 0.003–0.9 g·cm^−3^) and high internal surface areas (100–1200 m^2^·g^−1^), owing to their propensity to generate interconnected structures via sol–gel assembly approaches [1, 2, 3, 4, 5, 6, 7]. However, their intrinsic rigidity and susceptibility to brittle failure [8] under compressive loading severely restricts their practical applicability, with use limited to laboratory settings. This consequently hinders the adoption of aerogels in environmental remediation applications (e.g., reusable/cyclable oil or dye sorbents), soft/flexible thermal management materials (e.g., thermoregulative textiles), wearable electronics (e.g., strain sensors), and shock absorption. To overcome these limitations, polymer aerogels based on polyimide [9, 10, 11, 12] have been increasingly employed, to offer improved flexibility/softness. However, the thermoset nature of polyimide and similar polymer‐based aerogels offer limited recyclability and re‐processability. Alternatively, biopolymer‐based aerogels derived from natural sources, including cellulose derivatives [9, 10, 11], gelatin [12, 13], and chitosan [14] are among the first to have been implemented as sustainable alternatives to previous generations of aerogels. While these bio‐derived and compostable aerogels offer improved mechanical performance, their hydrophilicity limits usability, with fouling and moisture absorption causing property changes over time [8].

Among the most attractive biopolymers is the bio‐derived and industrially compostable poly(L‐lactide) (PLLA), which is a thermoplastic offering modest bulk modulus and strength (2–4 GPa and 60–80 MPa, respectively), as well as lower polarity compared to other natural biopolymers. While typically desired in its solid (i.e., nonporous) form, PLLA aerogels have been fabricated by numerous micro‐/nano‐structuring approaches for the purpose of lightweighting while imparting multifunctionality (e.g., hydrophobicity, thermal, or acoustic insulation behavior). Unfortunately, PLLA suffers from shortcomings including the low melting temperature of its thermally labile homocrystal (HC) polymorphs (< 180°C) and its intrinsic brittleness [15]. Moreover, it remains challenging to manipulate PLLA's crystal morphology and topology to yield aerogels with internal surface areas greater than ~ 90 m^2^·g^−1^ during sol–gel processing [16], making desirable functionalities like antifouling and water repellency (i.e., high water contact angles > 150°) in aerogels based solely on PLLA, inaccessible [17, 18].

However, from the inception of multicellular life on earth, natural evolution has yielded a confounding variety of biological materials and structures, meeting similar requirements for organisms to survive in their local environments, in a resource‐efficient manner. This has given rise to many naturally derived materials exhibiting such desirable combinations of mechanical toughness, water repellency, and/or thermal management performance, with favorably low material densities. A key attribute in developing this breadth of materials and structures arises from the formation of intricate assemblies of simple molecular building blocks (e.g., amino acids, sugars, minerals) into structures with features that span multiple length‐scales. In particular, certain plant leaf‐tissues like xylem, spongey/palisade mesophyll, epidermis, and cuticles exhibit structural hierarchy (e.g., cuticular tissue comprised by nano‐textured waxy epicuticular polymer crystals coating convex, micron‐scale cellular architectures) [19, 20, 21], which give rise to distinct material bulk properties that support specific organisms, imparting mechanical stability/defense, self‐cleaning ability, selective permeability, thermal regulation (e.g., via heat insulation/dissipation, UV–/visible light reflectivity and harvesting mechanisms), while being lightweight (e.g., imparting buoyancy) [19, 22]. Taking inspiration from such hierarchically structured plant‐tissues, emulating these structures in synthetic materials provides a path forward in developing new functional materials [23].

To overcome the challenges with creating green aerogels with reduced brittleness and improved usability, we adopt stereocomplexation as a design strategy to develop bio‐inspired structures, coupled with supercritical carbon dioxide drying (scCO_2_‐drying). This approach involves blending PLLA with its enantiomer poly(D‐lactide) (PDLA), yielding crystals comprised of both enantiomers, called “stereocomplex crystals” (SC)s. These SCs exist with a 3_1_ helical conformation that is more thermally stable than other PLA crystal polymorphs [24]. Specifically, SCs possess melting temperatures ranging from 200°C–230°C, in contrast to PLA HCs. Inclusion of SCs into a PLLA matrix is known to impart a dramatic increase in bulk modulus, strength, and toughness [25, 26, 27, 28], reduce hydrolysis rates [29], and increase PLA's heat deflection temperature by over 60°C [30], improving both its moisture resistance and thermal shape stability. It should be noted that SCs offer a variety of superstructure morphologies and topologies [27], making them a fascinating micro‐/nano‐structuring design tool. Beyond stereocomplexation, we apply scCO_2_‐drying for structural preservation of SC‐based gels. This drying approach offers lightweight, structurally homogeneous, and isotropic aerogels owing to the scCO_2_ reducing capillary stress on pore walls that otherwise leads to shrinkage/structural distortion [31, 32, 33].

Leveraging the morphological and topological variations possible in PLA SCs, we develop aerogels comprised of exclusively SCs, exhibiting network‐spherulitic morphology. This morphology yields aerogels that do not undergo brittle failure under compressive loading, and can resist cyclic loading without significant densification, making this material applicable to real‐life conditions. To tailor the stiffness of these aerogels, we draw inspiration from natural and synthetic hard/soft‐templated materials like certain plant tissues, freeze–dried starches, and polyurethane foams [34, 35], in SC‐based aerogels during sol–gel fabrication. We demonstrate that the morphology and bulk properties of these aerogels can be drastically altered by the implementation of polymer blending via incorporation of a so‐called “co‐gelling agent”, namely polycaprolactone (PCL); a compostable soft biopolymer that tailors material micro‐/nano‐morphology and functionality (e.g., tactile properties). Fundamentally, this reveals how the sequence of crystallization in multi‐polymer sol–gel systems governs the network morphology and topology in resulting gel derivatives.

Herein, we investigate the thermal phase transitions associated with gelation using differential scanning calorimetry (DSC), complemented by optical microscopy, and a detailed structure–property analysis of resulting aerogels, spanning the fine molecular to micro‐morphology length‐scales. Specifically, aerogel internal structures are characterized using scanning electron microscopy (SEM), Brunauer–Emmett–Teller (BET) theory, DSC, as well as temperature‐modulated differential scanning calorimetry (TM‐DSC), thermogravimetric analysis (TGA), and nanoindentation. These techniques evidence the formation of hierarchically porous aerogels comprised of interconnected spherulitic SCs; structural modification through PCL co‐gelation imparts variable degrees of phase separation, a consequence of gelation pathway and drying procedure. Fourier‐transform infrared spectroscopy (FTIR) was used to validate structural analyses, with 2D correlation analysis and synchrotron small angle x‐ray scattering (SAXS) applied to understand the configuration of aerogel constituents from the molecular scale.

Our newly developed aerogels offer tailorable modulus, do not undergo brittle failure under compressive loading (70% strain), and can exhibit minimal density change (< 15% increase) under cyclic compressive loading (100 cycles, 20% strain), characteristics resulting from their deformable hierarchically macro‐/meso‐porous internal structure. Beyond their tough mechanical performance, we identify that aerogels prepared through this pathway exhibit desirable characteristics including superhydrophobicity (water contact angle, ∼ 150°), great insulation (thermal conductivity, *k*_Cond_ ∼ 36 mW·m^−1^·K^−1^) and thermal management behavior, as well as intrinsically leakage‐free phase‐change insulation ability. These bulk characteristics, coupled with PLA's high thermal‐stability SCs as a structural template yield outstanding geometric–thermal stability, despite being comprised of thermally unstable constituents. Additionally, these aerogels are intrinsically superoleophilic and offer competitive performance in continuous oil–water separation (Flux ∼5881–17 352 L·m^−2^·h^−1^). The prepared aerogels are also recyclable/re‐processable by solution and melt‐processing owing to the curative‐ and additive‐free gelation approach applied during material preparation. Hence, this micro‐/nano‐structuring pathway is particularly useful in fabricating green, bio‐inspired materials for water repellent insulation, energy storage, and biomedical applications necessitating high surface area, soft porous monoliths.

## 2 Results and Discussion

### 2.1 Preparation of Multifunctional Aerogels

Aerogels were fabricated using a method highlighted in Figure 1A (detailed experimental description provided in Experimental section, and images of aerogels illustrated in Figure S1), with biopolymer pellets subject to thermally induced phase separation (TIPS) upon cooling during sol–gel processing, generating so‐called “thermogel” precursors. Calorimetric treatment was applied to thermogels to understand the structures that are implicated in TIPS, how they form, and whether these processes are thermally reversible (i.e., the class of gel formed), with calorimetric heating–cooling–heating thermograms illustrated in Figure 1B–D. It should be noted that regardless of gel composition analyzed, cooling results in a transformation from a transparent solution to an opaque gel, which is characteristic of a TIPS process forming microcrystalline superstructures.

**FIGURE 1:**
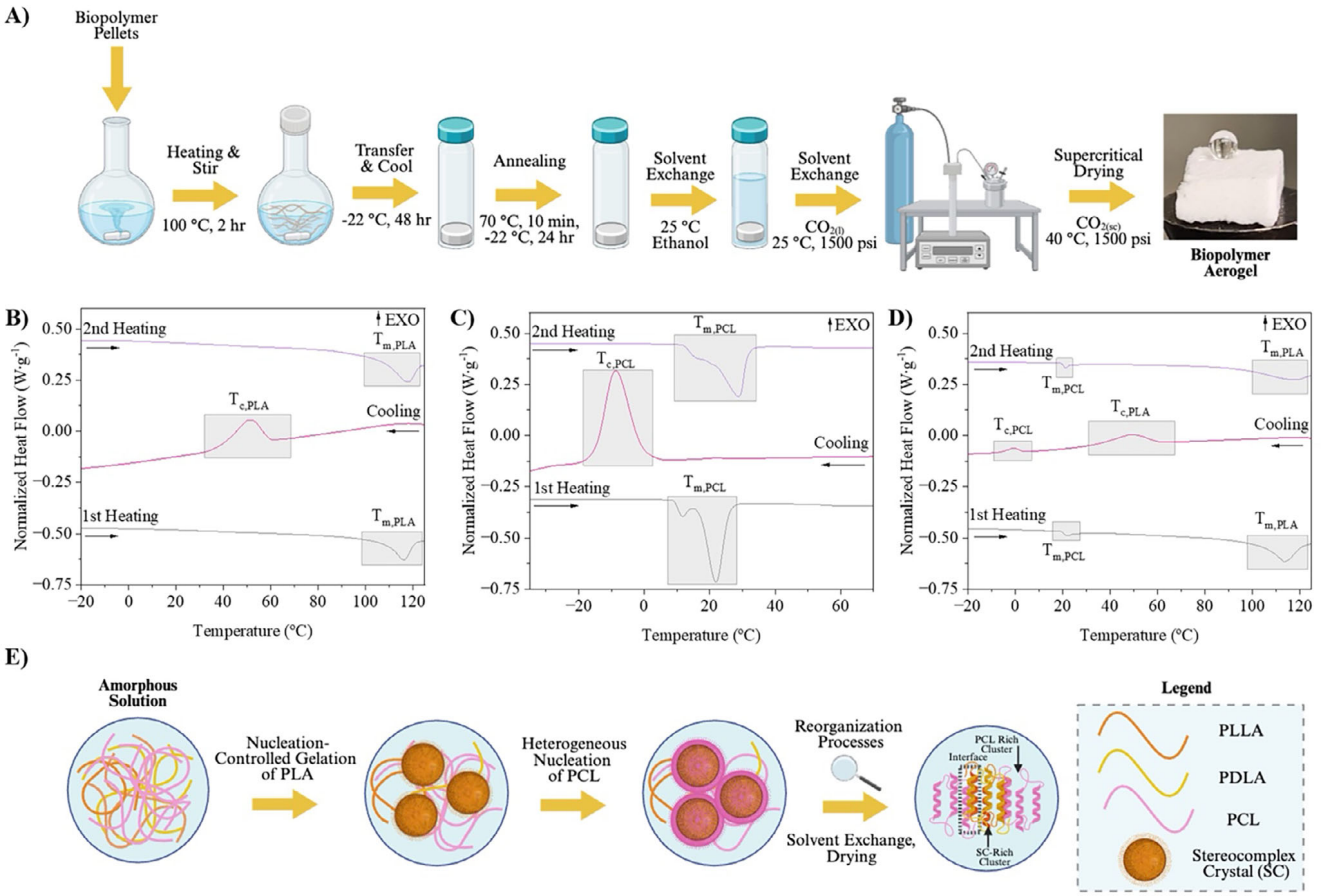
(A) Experimental sol–gel methodology used to fabricate the biopolymer aerogels in this work. Calorimetric heating, cooling, and re‐heating thermogram obtained for (B) 0 wt.% PCL thermogels (10.53 g PLA, 50/50 PLLA/PDLA ratio in 100 mL DMF; 10% of gel by mass is PLA), (C) 100 wt.% PCL thermogels (10.53 g PCL in 100 mL DMF; 10% of gel by mass is PCL). (D) 5 wt.% PCL blend thermogels (10.53 g polymer in 100 mL DMF; 10% of gel by mass is polymer, 5 wt.% of polymer fraction is PCL, the remainder is PLA with a 50/50 PLLA/PDLA ratio). (E) Schematic illustrating the proposed sequential gelation mechanism used to fabricate thermogels. Calorimetric thermograms are shifted by an arbitrary value for viewing clarity. Created in BioRender. Tuccitto, A. (2026) https://BioRender.com/v54zowq, Created in BioRender. Tuccitto, A. (2026) https://BioRender.com/c11tv9t.

To understand the thermal phase transitions associated with gel formation, we examine the degree of thermal hysteresis (i.e., *∆T*_Hyst_ = *T*_m_−*T*_c_) for PLA thermogels, PCL thermogels, and their blend thermogels (Figure 1B–D). In general, TIPS of semi‐crystalline polymers in sol–gel systems proceeds via either liquid–liquid de‐mixing (L → L) pathways like binodal or spinodal decomposition, or via liquid–solid de‐mixing pathways (L → C) like nucleation‐controlled crystallization or nanocrystal formation [36, 37, 38]. These phase separation processes can be later terminated by vitrification and/or crystallization processes with a greater degree of supercooling, imparting the resultant gel structure [39]. Generally, the presence of an exothermic phase transition upon cooling, combined with a large *∆T*_Hyst_, suggests a nucleation‐controlled crystallization process occurs during TIPS [40]. In contrast, minimal difference implies a spontaneous TIPS process (e.g., spinodal decomposition). Here, the phase transition inducing TIPS of the PLLA/PDLA control is exothermic, with the *∆T*_Hyst_ ≈ 65.8°C (Figure 1B). Similarly, the phase transition inducing TIPS upon cooling of PCL is also exothermic, but with a lower *∆T*_Hyst_ ≈ 30.7°C (Figure 1C). This is consistent with a greater kinetic barrier to self‐aggregation [41] for PLA than for PCL. This may be ascribed to the cooperative packing of PLLA and PDLA into the 3_1_ chain conformation needed for stereocomplexation. It should be noted that these mechanisms are a function of thermodynamic factors (e.g., polymer concentration in solution, blending temperature, cooling rate), as well as material properties (e.g., polymer *M*_w_ and *M*_n_, solvent type). While a wholistic analysis of process optimization is beyond the scope of this work, it is possible to alter this gelation mechanism through these means.

For thermograms corresponding to the 5 wt.% PCL blend thermogel (Figure 1D), two melting endotherms can be observed upon heating, two crystallization exotherms can be observed upon cooling, and two melting endotherms can be observed upon re‐heating, consistent with the melting and crystallization behaviors of the PLA and PCL thermogels. This suggests that PLA and PCL can both undergo thermoreversible crystallization‐associated phase transitions during cooling, with the presence of each blend constituent not adversely affecting the phase separation of the other blend constituent. We propose that upon cooling from the fully amorphous state, the PCL remains amorphous in the gel solution after crystallization of PLA, until a sufficiently large degree of supercooling is achieved for PCL crystallization. This behavior results from the notably lower melting temperature of PCL crystals in DMF compared to PLA crystals. Moreover, we observe a higher crystallization temperature (peak *T*_c_ *_, PCL_* of −0.77°C) for the 5 wt.% PCL blend thermogel compared to the PCL‐DMF thermogel (peak *T*_c, PCL_ of −8.76°C). This lower degree of supercooling required to induce crystallization of PCL suggests that the PCL may nucleate heterogeneously along the pre‐formed PLA crystalline structures, considering the sequential crystallization of the two materials. In contrast, the thermodynamic driving force for crystallization of PLA is largely unaffected by the presence of PCL, a probable result of the PCL remaining in its molten state during the self‐assembly of PLA.

To understand the role of PCL content on the crystal structure of prepared gels, calorimetric first heating thermograms for gels with varying PCL loading are provided in Figure S2. Here, the PLLA/PDLA control thermogel exhibits a clear endotherm at 116.3°C, corresponding to the fusion of PLA SCs, as can be seen in Figure 1B. This endotherm is in fact present for all analyzed thermogels, even in the presence of PCL. With the introduction of PCL at concentrations equal to or greater than 5 wt.%, there appears another endotherm near 22°C corresponding to the fusion of PCL crystals; this peak becomes even more apparent with 25 wt.% PCL. This observation aligns well with thermograms obtained for thermogels containing exclusively PCL and DMF (Figure 1C), as well as with the work of Von Rue et al. [42], who found that PCL‐DMF thermogels exhibit similar melting temperatures. Interestingly, macroscopic phase separation occurs during cooling when the PCL loading is increased to 50 wt.% of the polymer fraction, yielding gradient‐structured gels, and consequently, gradient‐structured aerogels (See images of aerogel and thermogel in Figures S1 and S3).

While there are numerous possible gelation pathways in polymer‐solvent systems during TIPS (see Figure S4 for possible pathways), a proposed mechanism for this sequential crystallization approach is illustrated schematically in Figure 1E. Upon cooling, the homogenized amorphous solvent‐polymer solution undergoes sequential phase separation, with PLLA and PDLA cooperatively crystallizing into a spherulitic network structure through a L → C pathway (see Figure S5 for microscopic evidence; polarized optical microscopy reveals a connected network of crystalline spherulites), followed by crystallization‐associated phase separation of PCL. While both PLA and PCL appear to undergo crystallization‐associated phase separation through nucleation‐controlled crystallization, the subsequent PCL phase assembly appears to be influenced by the pre‐formed SC structure, which may provide a structural template for subsequent crystallization of PCL (later evidenced in Figures 2, 4, and 5). These thermogels are precursors for aerogels, with their structures later tailored by reorganization and drying processes. Consequently, the structures formed during gel preparation directly govern the multitiered structural attributes of the resulting aerogels. Although DSC does not independently define the rheological gel point, the occurrence of crystallization upon cooling and the emergence of an interconnected crystalline morphology (observed by optical microscopy at the end of cooling) provides complementary evidence that gel formation is associated with crystallization. This interpretation is supported by the network‐spherulitic morphology retained in the dried aerogels, as discussed in subsequent sections.

**FIGURE 2:**
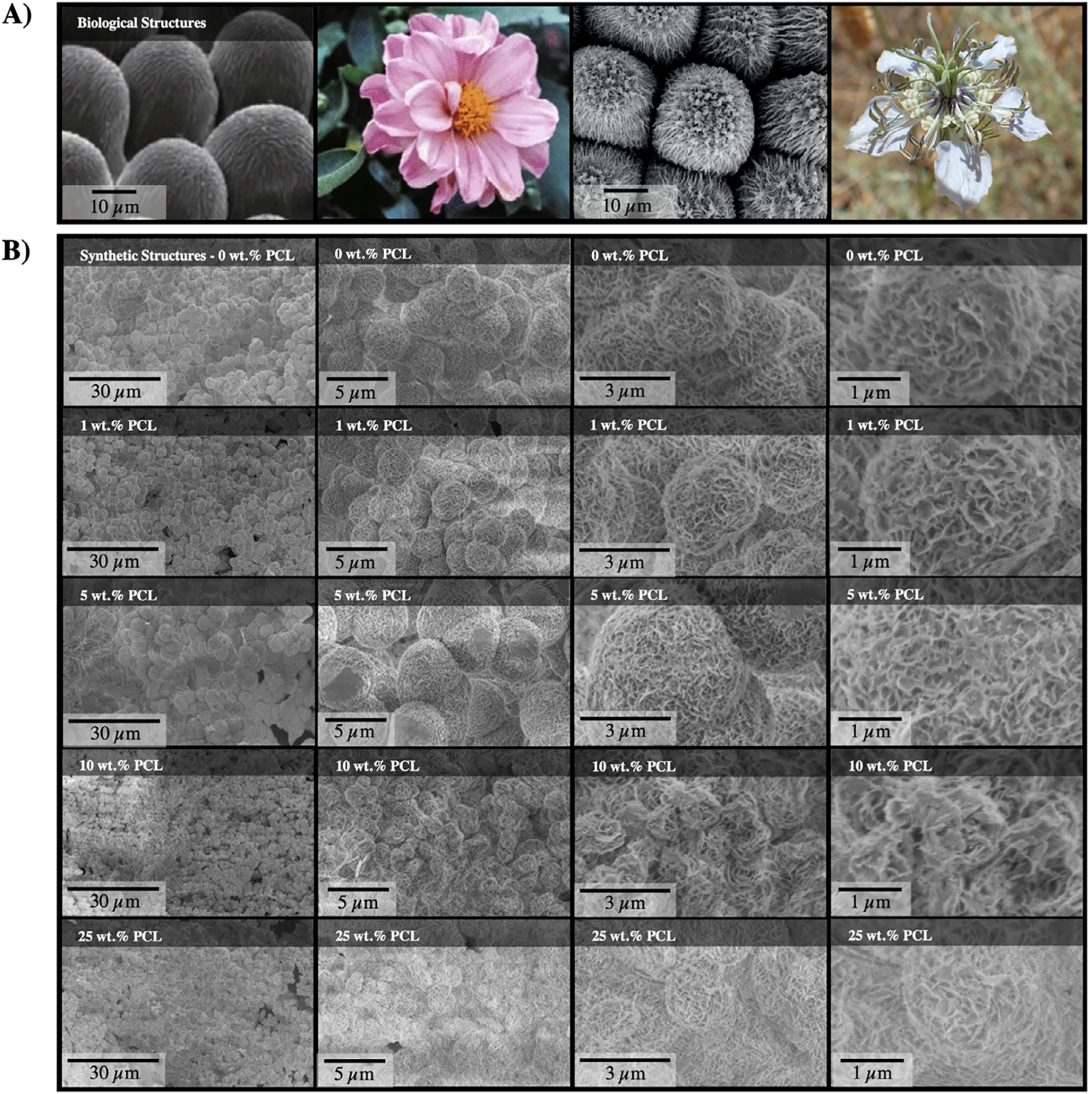
(A) Convex hierarchically structured cells of velvety *Dahlia* flower leaves (Left), and convex hierarchically structured cells of the *Nigella arvensis* petals (Right). Reproduced under terms of the CC‐BY license [19, 43]. Copyright 2017, adapted from Barthlott et al., published by Springer Nature. Reproduced under the terms of John Wiley and Sons [44]. Copyright 2019, adapted from Xu et al., published by Wiley. Reproduced under terms of the CC‐BY license [43, 45] (B) Morphology of 0, 1, 5, 10, and 25 wt.% PCL Aerogels. Images from left to right denote micrographs obtained at 5000, 20 000, 50 000, and 100 000× magnification.

### 2.2 Physical and Chemical Characterization of Aerogels

#### 2.2.1 Morphological and Topological Characterization of Aerogels

The envelope density and porosity of the prepared aerogels are reported in Table S1, with densities near 0.125 g·cm^−3^, and porosity values of nearly 90%. Scanning electron micrographs of aerogels with PCL loadings ranging from 0–25 wt.% are shown in Figure 2. These aerogels are internally isotropic and exhibit good structural homogeneity, regardless of composition. The PLLA/PDLA control aerogel exhibits a structure comprised of discrete spherulites possessing isotropic texture, with particle size distributions illustrated in Figure S6. With PCL loadings of 0, 1, and 5 wt.%, these crystal structures are 2.71 ± 0.37, 2.76 ± 0.28, and 4.78 ± 0.37 µm, with some structural distortion toward more globular structures as the PCL loading is increased. In contrast, with 10 and 25 wt.% PCL, the textured globule size decreases to 1.59 ± 0.21 and 2.08 ± 0.34 µm, respectively, and becomes more continuous.

The hierarchically structured aerogels fabricated possess micron‐scale spherulites, and nano‐scale texture, inspired by plant cuticular tissues. While the cuticular structure varies depending on the plant class, species like *Dahlia* and *Nigella arvensis* possess similar micro‐/nano‐morphologies. Specifically, their cuticular tissue exhibit nano‐textured epicuticular polymer crystals coating convex, micron‐scale cellular architectures (Figure 2A). Moreover, sequential crystallization provides a means of controlling the convexity of the spherulites (i.e., shape/aspect ratio), with crystallization of the PCL along the SC giving rise to diversity in surface feature shapes, akin to the variety of plant cuticular cells reported [19].

In comparison to existing literature, the morphologies obtained in this work differ drastically. While others have reported a variety of SC‐containing aerogels and monoliths with a broad range of morphologies including various types of network and fibril structures [17, 18, 46, 47, 48], the network‐spherulitic structures obtained in this work differ distinctly and are obtained in the absence of any additives that could detriment the green nature of the selected aerogel constituents. It is known that PLA SCs can exhibit morphological and topological variations depending on material‐related factors including molecular weight, chain architecture, enantiomer ratio, optical purity, and process‐related factors including blending and crystallization conditions (e.g., temperature/cooling rate, solvent type, and polymer‐solvent ratio) that alter the crystal growth mechanism (i.e., local chain packing instabilities along the SC growth front) [27]. However, spherulites are among the highest‐order SC superstructures that can form. In this case, the development of a network‐spherulitic structure is generated in monoliths, regardless of drying procedure applied (see Figure S7 for SEM illustrating micro‐/nano‐structure of freeze–dried PLLA/PDLA monoliths), suggesting that this crystalline superstructure morphology and topology result from TIPS, in which liquid–solid de‐mixing drives the isotropic growth and organization of well‐ordered lamellar structures into spherulitic superstructures, until crystal impingement occurs. Notably, the network‐spherulitic morphology and topology are retained in the presence of PCL. This structural retention is consistent with the sequential crystallization approach, in which PLA self‐assembles into crystalline superstructures prior to subsequent crystallization of PCL.

#### 2.2.2 Pore Structure and Crystallinity Analysis

Nitrogen adsorption–desorption isotherms were obtained to probe the fine pore structure of the prepared aerogels. From Figure 3A, prepared aerogels exhibit a Type IV adsorption isotherm regardless of PCL loading, indicative of a mesoporous structure (2–50 nm). This is validated by pore size‐distributions illustrated in Figure 3B and cumulative pore volume estimates in Figure 3C, which reveal narrow pore width distributions with peak pore widths centered between 7 and 9 nm. Beyond pore width distribution, the BET method was used to estimate the specific surface area of aerogels (*S*_BET_). Here, *S*_BET_ was estimated as 175 m^2^·g^−1^ in the absence of PCL, which changes to 186, 168, 110, and 63 m^2^·g^−1^ with 1, 5, 10, and 25 wt.% PCL, respectively. Interestingly, the presence of < 10 wt.% PCL imparts among the highest *S*_BET_ for SC‐based PLA aerogels [18]; this is ascribed to the structural retention imparted through scCO_2_‐drying that suppresses the collapse of pores. In contrast, the application of freeze–drying appears to result in the collapse of pores, leading to a reduction in the internal surface area (*S*_BET_ of 32.8 m^2^·g^−1^).

**FIGURE 3:**
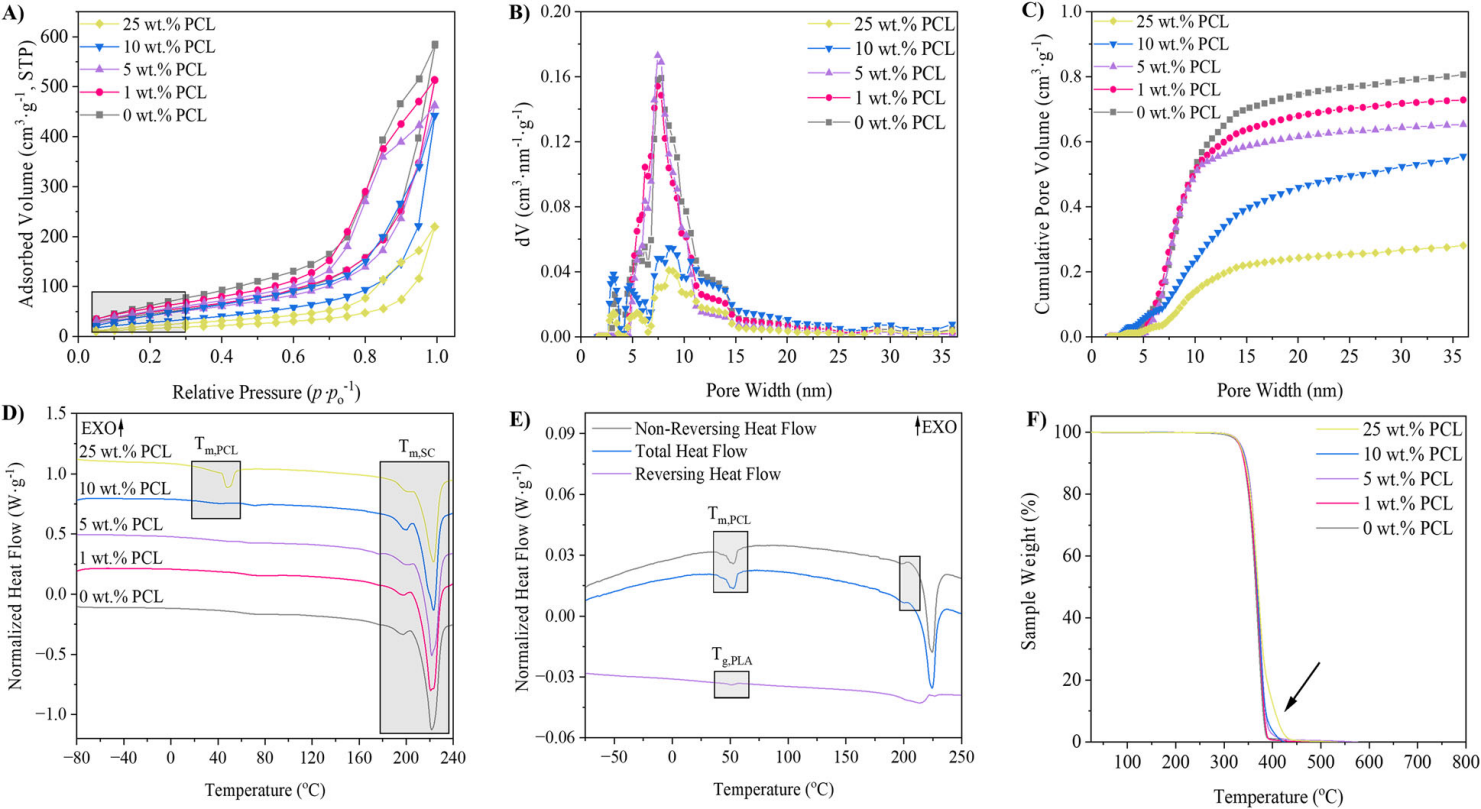
(A) Nitrogen adsorption–desorption isotherms, (B) pore size distribution, (C) cumulative pore volume with respect to pore width, (D) DSC heating thermograms of prepared aerogels obtained using a heating rate of 10°C·min^−1^, (E) TM‐DSC heat–cool thermograms illustrating the reversing, non‐reversing, and total heat flow with respect to temperature obtained for the 25 wt.% PCL aerogel. (F) Thermogravimetric analysis illustrating the weight loss of aerogels with respect to temperature under heating from 25°C to 800°C, at a rate of 10^⚬^C·min^−1^. Calorimetric thermograms are shifted by an arbitrary value for viewing clarity.

The bulk properties of polymers and their derivatives are directly attributed to material micro‐/nano‐structures and thermal properties. As such, DSC was used to quantify critical thermal properties including aerogel constituent glass transition temperature (*T*_g_) and degree of crystallinity (***χ***_c_). Additionally, DSC analysis was used to identify crystal polymorphism in prepared aerogels, with quantitative analysis provided in Table S2. DSC heating thermograms illustrating each aerogel's properties can be found in Figure 3D (enlarged melting endotherms of PLA found in Figure S8). From calorimetric analysis, the PLLA/PDLA control with 0 wt.% PCL exhibits a *T*_g_ of 60.1°C, and a bimodal melting endotherm above 180°C. While the melting endotherm being above 180°C suggests the formation of exclusively SCs, the bimodal melting endotherm is consistent with the formation of a network‐spherulitic crystal topology [28, 49, 50], as was observed microscopically. Specifically, the high‐melting temperature (*T*_m_,_high_) peak is believed to correspond to the fusion of the higher‐order spherulitic structures as were evidenced via microscopic analysis. In contrast, the lower melting temperature (*T*_m, low_) crystal population likely corresponds to the more thermally labile interspherulitic regions (i.e., grain boundaries or regions connecting spherulites). This is consistent with similar observations by Saeidlou et al. [49].

With the inclusion of 1 wt.% PCL, the thermal properties of the aerogels change minimally relative to the PLLA/PDLA aerogel control. However, with the inclusion of 5 wt.% PCL, a small endotherm, which overlaps with PLA's *T*_g_ can be observed, corresponding to the fusion of the PCL crystals. Generally, it is believed that PCL crystals do not exhibit polymorphism owing to the absence of pendant or side‐chain that would otherwise permit multiple metastable chain packing configurations, and exists with an orthorhombic unit cell [51]. With the inclusion of 25 wt.% PCL, the PCL melting endotherm becomes more pronounced with its peak observable at 48.0°C, but convoluted with *T*_g, PLA_. The development of a discernable melting endotherm for the PCL indicates the occurrence of noticeable phase‐separation between PLA and PCL. We believe that with elevated PCL content, phase separation occurs during gel preparation, likely owing to saturation of the PLA template's surface. Plausibly, PCL chains that are unable to nucleate at the PLA SC interface are consequently forced to self‐aggregate into distinct, phase‐separated crystalline domains, thereby minimizing their interactions with the solvent. This is reflected in the reduction in *S*_BET_ with increasing PCL content beyond 1 wt.%, as well as the increasing degree of crystallinity with increasing PCL content (see Table S2).

To deconvolute these overlapping thermal events evidenced in calorimetric heating profiles and validate crystallinity estimates, the heating rates during calorimetric analysis were varied from 5–40°C·min^−1^, given that devitrification processes are typically a stronger function of heating rate than fusion processes, as evidenced in Figure S9. Although these thermal phenomena appear heavily convoluted, even at more rapid heating rates, we apply TM‐DSC analysis to better resolve these thermal phenomena. A detailed interpretation of TM‐DSC analysis can be found in the Supporting Information and Figure S10. Based on this analysis, it appears that the presence of PCL at all concentrations tested does not adversely affect the degree of SC crystallinity (*χ*_SC_) in prepared aerogels. The *χ*_SC_ of aerogels prepared by scCO_2_‐drying are estimated as 59.7%, 60.3%, 61.5%, 61.9%, and 62.6%, with PCL contents of 0, 1, 5, 10, and 25 wt.%, respectively. To the best of our knowledge, these *χ*_SC_ values are among the highest obtained in literature for PLLA/PDLA aerogels. This high *χ*_SC_ is a consequence of the sequential crystallization strategy applied, with crystallization of PLA occurring independently prior to the crystallization of PCL. Specifically, while PLA undergoes crystallization during sol–gel processing, PCL remains amorphous until a greater degree of supercooling is achieved, as was illustrated via calorimetric analysis of thermogels in Figure 1. As such, the self‐aggregation of PLA into ordered crystalline superstructures is largely uninhibited by the presence of such amorphous PCL chains, a result of the blending temperature being much greater than *T*_m, PCL_.

TGA was conducted to quantify the thermal stability of the blend aerogels, with thermogravimetric behavior of aerogels illustrated in Figure 3F, as well as corresponding derivative plots and thermogravimetric behavior of the neat aerogel constituents shown in Figure S11. Additionally, the test temperature corresponding to 5%, 50%, 75%, and 90% weight loss are denoted in Table S3 as *T*_5%_, *T*_50%_, *T*_75%_, and *T*_90%_, respectively. The neat PLLA and PDLA exhibit similar degradation behavior, undergoing sharp, single‐step degradation with *T*_50%_ of nearly 350°C–357°C, owing to non‐radical backbiting ester interchange [52]. As well, the PLLA/PDLA control aerogel exhibits a higher *T*_50%_ of 365.0°C, which may be ascribed to the thermal stability of the SCs relative to either enantiomeric counterpart, as is consistent with other work [18]. This behavior likely results from the 3_1_ helical chain conformation of SCs, which reduces the susceptibility of PLA chains to backbiting degradation mechanisms owing to effective “shielding” of its ester groups within the SC lattices. In contrast, despite its lower melting temperature, PCL is known to degrade at a much higher degradation temperature (*T*_50%_ of 392.4°C) [52, 53, 54]. As such, it is expected that inclusion of PCL in PLA‐dominant aerogels would increase noticeably the degradation temperature. Interestingly, the *T*_50%_ in all PCL‐containing aerogels increases by at most 5°C (25 wt.% PCL) relative to the control aerogel, implying a high degree of interaction between the PLA and PCL. However, the *T*_75%_ and *T*_90%_ are nearly 10°C and 25°C greater in the 25 wt.% PCL aerogels than in the PLA control, implying considerable phase‐separation. Specifically, while phase‐mixed PCL localized near PLA chains degrade homogeneously, the phase‐separated PCL chains degrade at distinctly higher temperatures. This interpretation is supported by the shoulder that emerges in the derivative weight loss plot with increasing PCL loading (Figure S11), and with phase‐separation confirmed by the appearance of a distinct melting endotherm in calorimetric heating curves (Figure 3D) with 25 wt.% PCL.

Nanoindentation with SPM imaging was further employed to quantify the stiffness (reduced modulus, *E*_r_) of the spherulitic structures and to elucidate the influence of PCL addition on the material local stiffness (Figure S12). A description of *E*_r_ and its measurement method can be found in the Supporting Information. The PLLA/PDLA sample exhibited an *E*_r_ of 4.77 ± 0.32 GPa, whereas the 5 wt.% PCL sample showed an *E*_r_ of 3.61 ± 0.28 GPa. Notably, the incorporation of 5 wt.% PCL results in a measurable decrease in stiffness, which can be rationalized based on the sequence of gel constituent crystallization. Since the stiffer PLA phase undergoes crystallization prior to the softer PCL phase, the latter subsequently crystallizes as highly textured lamellae associated with the pre‐existing PLA domains. Thus, the presence of softer PCL‐rich regions localized near the PLA domains diminishes the overall rigidity of the spherulitic superstructure, leading to lower stiffness values.

#### 2.2.3 FTIR Spectroscopy Analysis and Synchrotron Small Angle X‐Ray Scattering

FTIR spectroscopy was conducted on the prepared aerogels to understand the driving forces that promote the formation of this crystal assembly (i.e., discern covalent/hydrogen bonding interactions), as well as a complementary technique to DSC to confirm the existence of exclusively SCs. Full FTIR spectra of the prepared aerogels are illustrated in Figure S13, with distinct spectral regions illustrated in Figure 4. Additionally, a detailed spectral assignment table has been provided in Table S4. First, it should be noted that there is no detectable band corresponding to O─H stretching, likely because of the trace amount of chain ends, which may be considered negligible owing to the high *M*_W_ material grades used [55].

**FIGURE 4:**
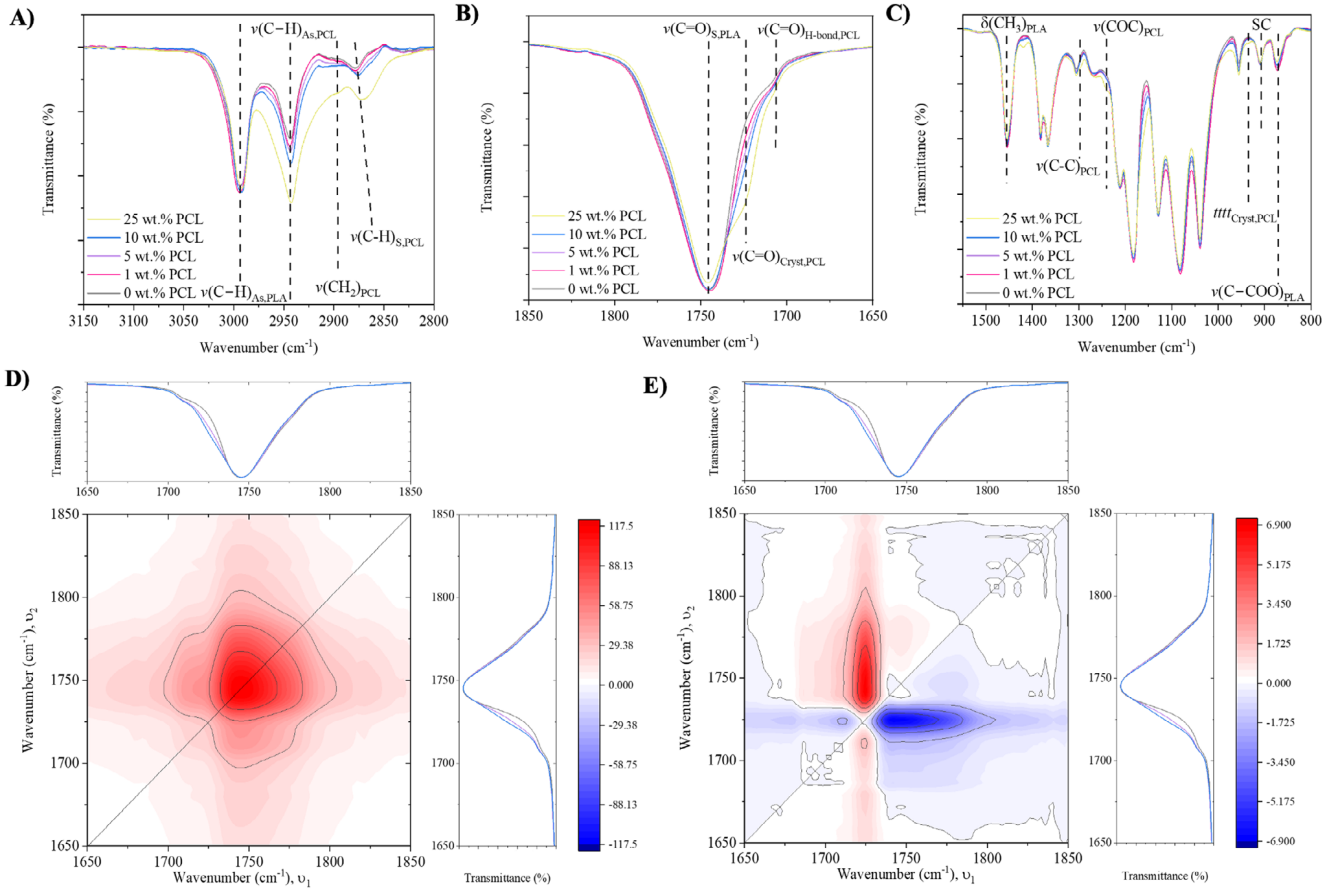
FTIR spectra obtained for scCO_2_‐dried aerogels over distinct spectral wavenumber regions, including (A) 3150–2800, (B) 1850–1650, and (C) 1550–800 cm^−1^. Two‐dimensional correlation analysis is applied to the spectral range of 1850–1650 cm^−1^, with (D) synchronous, and (E) asynchronous spectra reported.

FTIR spectra of the scCO_2_‐dried aerogels with varying PCL loadings, illustrating bands between 3150 and 2800 cm^−1^, can be found in Figure 4A. Spectral bands can be observed at 2993, 2943, 2895, and near 2877–2866 cm^−1^, corresponding to certain C─H stretching modes of PLA and PCL [56, 57, 58, 59, 60], other C─H stretching modes of PLA and PCL [56, 59, 61, 62, 63]. C─H stretching of PCL [64], and symmetric C─H stretching of methylene groups [61, 63]. Here, the intensity of the C─H stretching band near 2993 cm^−1^ appears independent of PCL content, while the C─H stretching band near 2943 cm^−1^ shows a clear increase in intensity with increasing PCL loading, likely attributed to the abundant methylene groups in PCL. In fact, this band has been previously ascribed to the methylene groups adjacent to PCL's ester group's oxygen atom, specifically within crystalline structures [64]. This thereby implies some degree of crystallinity in the PCL phase that becomes more pronounced for the 25 wt.% PCL aerogel, owing to the crystallization of phase‐separated PCL domains. Similar behavior was observed for the band near 2985 cm^−1^, which corresponds to the methylene group bonded to the carbonyl ester [64]. Following a similar trend, the band near 2877–2866 cm^−1^ that presumably corresponds to the C─H stretching of methylene groups that do not directly neighbor the PCL's ester groups, appears to decrease in wavenumber to 2866 cm^−1^ with increasing PCL loading. In contrast, for the PLLA/PDLA control aerogel, this band likely corresponds to the ─CH methine group of PLA [65].

Beyond these, Figure 4B shows the absorption bands associated with C═O stretching of the prepared aerogels between 1850 and 1650 cm^−1^; bond stretching modes in this region are particularly sensitive to environmental changes (e.g., hydrogen bonding variations, entrapment in crystal lattices). The PLLA/PDLA control aerogel exhibits a symmetric C═O stretching band centered near 1745 cm^−1^ [65]. This is consistent with the *trans–trans* (*tt*) chain conformation characteristic of the 3_1_ *tt* helical conformation of PLA SCs [65]. Interestingly, with increasing PCL loading, a series of distinct stretching bands arise at 1723, 1705, and 1688 cm^−1^, respectively, that become obvious with 25 wt.% PCL. The band near 1723 cm^−1^ has previously been ascribed to the C═O stretching of crystalline PCL [66, 67], while the band near 1705 cm^−1^ has been ascribed to C═O groups in amorphous PCL that are hydrogen bonded [68, 69, 70]. The band near 1723 cm^−1^ appears to increase noticeably in intensity with 25 wt.% PCL and corroborates observations from calorimetric analysis that illustrate the fusion of phase‐separated PCL crystals. From this spectral region, it can be deduced that as the PCL content increases in aerogels, the PCL chains become distinctly phase‐separated, a likely result of crystallization that expels the PCL to the PLA‐phase's interspherulitic regions.

Additionally, FTIR spectra of prepared aerogels in the range of 1550–800 cm^−1^ can be found in Figure 4C. Here, a band is present at 908 cm^−1^ that has previously been ascribed to the 3_1_ helical chain conformation of PLA SCs, which can be observed for all aerogels prepared [65]. Interestingly, the band near 923 cm^−1^ that has been previously ascribed to the presence of α‐crystal helices of PLA cannot be observed, supporting the exclusive existence of SCs in the PLA phase of prepared aerogels, further supporting our calorimetric analysis. Additionally, the band present at 1039 cm^−1^ is observed and has been previously attributed to the ─C─CH_3_ segment of the PLA SCs [65].

To support our hypotheses regarding phase separation of PLA and PCL, we apply 2D correlation analysis to the FTIR spectroscopic data. Such 2D correlation analysis was previously generalized by Noda et al. [71]. Details regarding the applied 2D correlation transforms and their interpretation can be found in Supporting Information. Based on this analysis, an increasing PCL loading in aerogels is believed to result in the saturation of the SC interspherulitic region with PCL, followed by phase separation of PCL into distinct crystalline domains.

Additionally, Synchrotron SAXS was conducted on prepared aerogels to provide a clearer understanding of the configuration of the crystal structures comprising the PLLA/PDLA control aerogel and understanding how PCL inclusion alters the morphology/topology of these aerogels. The evolution of a new peak at *q* ∼ 0.34 Å^−1^ was observed with increasing PCL content [72]. (Figure 5A, inset ii), consistent with the previously observed *χ_c, PCL_* (Table S2). The aerogels also had broad correlation peaks in the region of ∼0.06–0.135 Å^−1^ (Figure 5A, inset i) which can be fitted using the Teubner–Strey (T–S) model. This model has been previously demonstrated as an appropriate method to analyze PLLA/PDLA SC inter‐cluster distances [73]. T–S model (Equations S4 and S5) fitting yielded SC inter‐cluster distances between 8.70 and 9.73 nm (Tables 1 and S5 for T–S fitting parameters) which showed strong agreement with the observed average pore widths (Figure 3B), suggesting the mesoporosity of the aerogels evidenced in Figure 3B may result from the space between the SC‐rich clusters.

**FIGURE 5:**
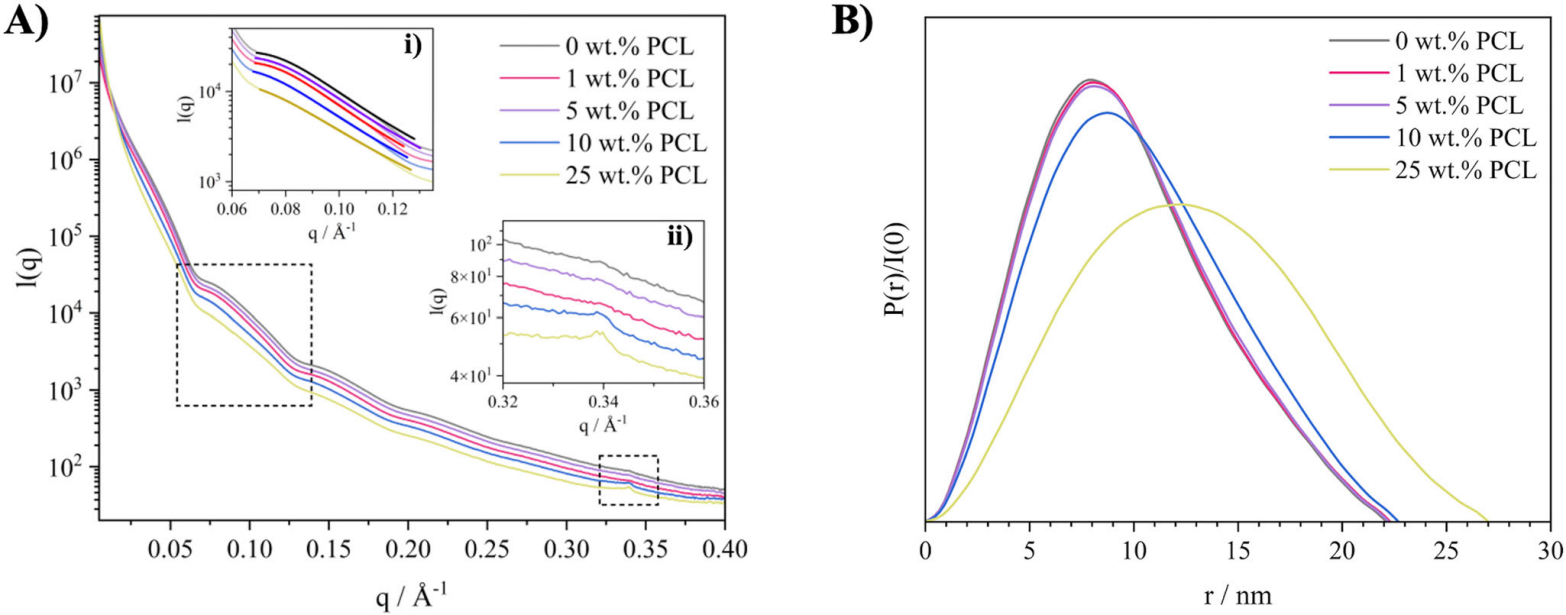
Synchrotron SAXS data illustrating (A) intensity (*I*(*q*)) with respect to the scattering vector (*q*), with the Teubner–Strey (T–S) fit of the broad peak corresponding to PLA SCs (inset i) highlighted, and the peak corresponding to crystalline PCL (inset ii), as well as (B) the corresponding *P*(*r*) plots (i.e., pair distance distribution function).

**TABLE 1:** Calculated T–S inter‐cluster distance, radius of gyration, and maximum inter‐cluster distance from T–S and *P*(*r*) fittings.

Sample name	T–S inter‐cluster distance (nm)	*R_g_* (nm)	*D_max_* (nm)
0 wt.% PCL	8.70	7.36	22.1
1 wt.% PCL	8.82	7.42	22.29
5 wt.% PCL	8.81	7.45	22.21
10 wt.% PCL	9.39	7.83	22.68
25 wt.% PCL	9.73	9.81	26.99

The SC inter‐cluster distances showed relatively little variation with increasing PCL content, indicating that the characteristic spacing of the SC‐rich domains is insensitive to PCL incorporation. In contrast, both *R*_g_ and *D*_max_ (Table 1), determined from the *P*(*r*) fitting of the SAXS plots, exhibited relatively constant nanodomain sizes at low PCL content (0–5 wt.%), whereas higher PCL contents (10–25 wt.%) resulted in increases in both average and maximum nanodomain sizes. These observations are consistent with the progressive structural development around the pre‐existing SC‐rich domains during sequential crystallization, wherein increasing PCL availability may promote the development of PCL‐rich lamellar structures localized near the pre‐existing SC‐rich domains.

### 2.3 Compression Testing

Stress–strain curves illustrating the compressive behavior of scCO_2_‐dried aerogels are illustrated in Figure 6A with aerogels subject to up to 70% strain. As well, compressive stress‐strain curves corresponding to freeze‐dried monoliths (cryogels) are provided in Figure S14. Interestingly, the freeze–dried monolith suffers from catastrophic failure at < 10% strain, while the scCO_2_‐dried PLLA/PDLA control aerogel does not undergo catastrophic failure when even subject to 70% compressive strain. This difference in behavior can be ascribed to several key microstructural attributes. First, in the freeze–dried sample, SCs appear as fused spherulites (Figure S7), in contrast to scCO_2_‐dried aerogels that exhibit discrete spherulites. Consequently, the freeze–dried monolith exhibits a lower interfacial area in conjunction with lower microstructural uniformity. This contributes to a heterogeneous stress distribution which leads to its catastrophic failure. In contrast, the scCO_2_‐drying imparts an isotropic SC network that fosters homogeneous stress distribution which leads to a tougher mechanical response. Finally, the SCs existing as discrete isotropic textured spherulites in the scCO_2_‐dried aerogel microstructure circumvents edge effects that characteristically concentrate stress and initiate fracture [74]. These behaviors are evidenced by fractography analysis of the PLLA/PDLA control after compression to 70% strain, as is illustrated in Figure 6D, which shows the absence of catastrophic cracks. It should be noted that for bending‐dominated lattice structures including open‐cell foams and aerogels, the material undergoes a series of failure modes including: 1) linear elastic (lattice bending regime), until it reaches its elastic limit, followed by 2) buckling (i.e., cell edge yielding, plastic deformation at the plateau stress), and later 3) densification (i.e., where opposing sides of cell walls contact) [74, 75]. For the scCO_2_‐dried aerogel, even after densification, the aerogel microstructure does not show developed crack propagation. Rather, there is noticeable fusion of neighboring SCs under high compressive strain (70%). This unusual behavior can possibly be attributed to interpenetration of neighboring crystals, as there is no clear boundary between the amorphous and crystalline phases comprising the SCs, allowing interfusion of the supramolecular architecture. Consequently, during the densification phase, this architecture leads to a tougher and more uniform response preventing catastrophic failure. Unexpectedly, even after compression to this extent, the crystals comprising the aerogel exhibit obvious nano‐scale texture.

**FIGURE 6:**
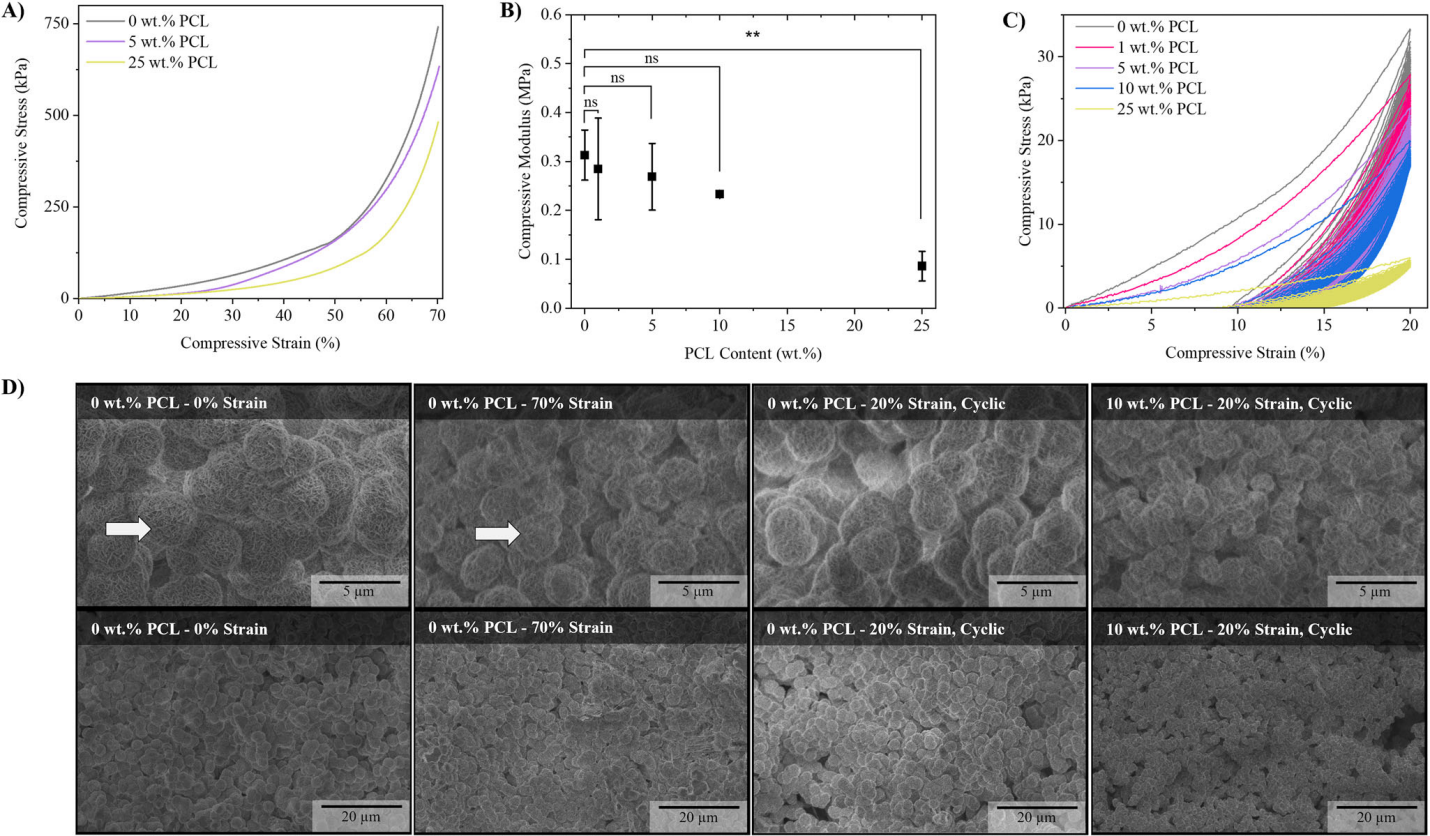
(A) Compressive stress–strain behavior of select aerogels subject to 70% strain, (B) compressive modulus obtained from compression to 20% strain, with respect to PCL loading. (C) Stress–strain curves obtained for aerogels under cyclic compression testing (20% compressive strain) over 100 cycles. (D) Scanning electron micrographs of select aerogels before and after compressive testing, obtained at 20 000 × (top row) and 5000 × (bottom row) magnification. Arrows denote nanoscale texture that is maintained after compressive testing. Data labels of ns, *, **, ***, **** represent significance levels of *p* > 0.05, *p* < 0.05, *p* < 0.01, *p* < 0.001, and *p* < 0.0001, respectively, obtained from Dunnett's multiple comparisons test.

This desirable failure mode is maintained in scCO_2_‐dried aerogels with the inclusion of PCL. For scCO_2_‐dried aerogels, compressive modulus values ranged from 0.313 ± 0.051 to 0.086 ± 0.030 MPa with PCL loadings of 0 and 25 wt.%, respectively (see Figure 6B). While the compressive modulus exhibits no statistically significant change with PCL loadings up to 10 wt.% PCL, contents above this threshold result in softer aerogels, especially when compared to those of similar envelope densities (Figure S15). Regardless of PCL loading, tested aerogels do not undergo brittle failure, even when subject to high compressive strains up to 70%. While in literature, PLA/PCL blends typically suffer from interfacial debonding under stress [76, 77], which results in abrupt catastrophic failure [78], the PLA/PCL interfaces developed in this study do not suffer from this behavior. Hence, co‐gelation modification using PCL can produce softer aerogels without compromising their ability to undergo high compressive strain without abrupt failure. Mechanistically the reduction in modulus can be regarded as a result of inclusion of the softer PCL phase forming a more continuous structure, in aerogels comprised of the more rigid PLA SCs, evidenced by nanoindentation.

Additionally, scCO_2_‐dried aerogels were subject to cyclic compression tests over 100 cycles at a fixed strain of 20%. Representative cyclic compression curves are illustrated in Figure 6C, demonstrating the stress–strain behavior of the aerogels under cyclic loading. The inclusion of PCL did not adversely affect the ability of the aerogels to resist cyclic loading without significant densification (Table S7). This is ascribed to the presence of the isotropic and uniform, network‐spherulitic structure within all prepared aerogels, and the nanomorphology of the blend aerogels with PCL. In blend aerogels, our microscopic analysis revealed this material configuration can accommodate the aerogel cell lattice bending, buckling, and densification modes when strained, without detectable cracks via interfacial de‐bonding that are typically apparent in blends of PLA with PCL [76].

### 2.4 Functional Property Analysis

#### 2.4.1 Thermal Management Performance and Thermal Stability

The thermal conductivity (*k*_Cond_) and porosity of prepared aerogels with respect to PCL content are illustrated in Figure 7. The PLLA/PDLA control exhibits excellent insulation behavior with an average thermal conductivity of 36.1 ± 2.1 mW·m^−1^·K^−1^. With 1, 5, 10, and 25 wt.% PCL, the thermal conductivity values are 37.6 ± 3.0, 39.5 ± 3.8, 36.9 ± 1.6, and 36.6 ± 1.1 mW·m^−1^·K^−1^, respectively, with no statistically significant variations in *k*_Cond_ with increasing PCL loading. This occurs likely owing to the similar porosity values among the prepared aerogels. Beyond their thermal insulation ability, the inclusion of a low melting‐temperature component into aerogels imparts the potential for thermoregulative behavior. Specifically, such so‐called phase‐change insulators (PCIs) have been recently sought for passive cooling applications, owing to their ability to dynamically absorb and release heat to and from the environment via phase changes. For building insulation applications, phase transformations centered near room temperature yield the most effective performance. Moreover, it is desirable to combine the benefits of PCIs in regulating environmental temperatures with strong thermal insulators [79]. We identify the prepared blend aerogels in this work as great candidates for PCIs owing to both the competitive insulation ability and the thermal phase‐change effect associated with the low onset and peak *T*_m_ of the blend aerogel's PCL phase. While polyethylene glycol (PEG) is commonly used as the PCI in many other materials, its tendency to leak when molten [80], as well as its hydrophilic nature and resulting moisture retention makes these materials unsuitable for PCI applications. Moreover, the compostable nature of PCL makes this a green alternative.

**FIGURE 7:**
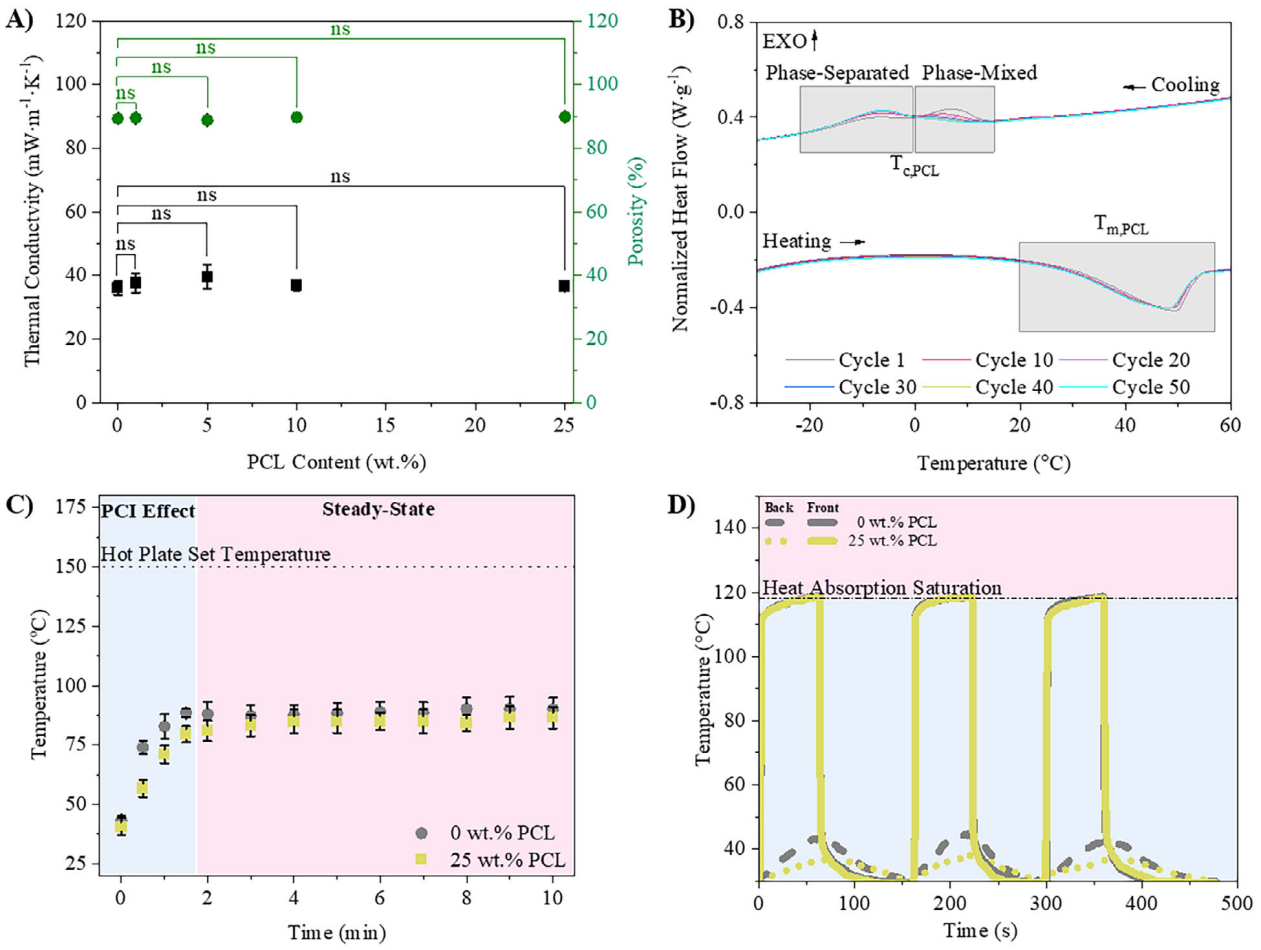
(A) Thermal conductivity and porosity with respect to PCL loading of prepared aerogels, (B) heating and cooling thermograms obtained from cyclic calorimetric profile, with constant linear heating rate of 20^⚬^C·min^−1^ and cooling rates of 10^⚬^C·min^−1^. Calorimetric data has been shifted by an arbitrary factor for viewing clarity. (C) Temperature of 0 and 25 wt.% PCL aerogel on hot plate with respect to time, as obtained from thermal camera measurements. (D) Temperature of 0 and 25 wt.% PCL aerogel under radiative heating, with respect to time. Data labels of ns, *, **, ***, **** represent significance levels of *p* > 0.05, *p* < 0.05, *p* < 0.01, *p* < 0.001, and *p* < 0.0001, respectively, obtained from Dunnett's multiple comparisons test.

To understand the heat absorption and release behavior of these materials, the prepared aerogels are heated from –50°C to 80°C (i.e., PLA phase is not affected) at a rate of 20°C·min^−1^, followed by cooling at 10°C·min^−1^ to –50°C, with 50 heat–cool cycles performed via DSC as has been conducted in other studies [81, 82]. Upon heating, an endotherm can be observed consistently near ∼ 48°C corresponding to the fusion of PCL, similar to Figure 3D. Upon cooling, a bimodal exotherm can be observed between –20°C and 10°C, corresponding to the subsequent crystallization of PCL. Specifically, when PCL is localized near PLA, PCL crystallization is facilitated by heterogeneous nucleation along the PLA phase. Consequently, consecutive heat–cool treatment leads to the progressive phase‐separation of PCL initially localized near the PLA phase, followed by the formation of distinct PCL‐rich crystalline domains. The higher and lower crystallization temperature modality (near 7°C and –7°C) of the peak likely corresponds to the crystallization of interface‐localized and phase‐separated regions of PCL.

Thermal insulation and thermal management evaluation system schematics are provided in Figure S16, with thermographic images exhibiting the thermal shape‐stability of these aerogels under high temperature found in Figures S17 and S18. Moreover, measured temperatures have been extracted and are shown in Figure 7. In thermal insulation performance evaluation, the aerogels are placed on a hot plate heated to 150°C, with thermographic images of the top surface obtained in 30 s or 1 min intervals over 10 min. Both the PLLA/PDLA control aerogel and aerogel containing 25 wt.% PCL exhibit great thermal insulation and thermal stability, with no obvious geometric changes (e.g., shrinkage/relaxation, warping) occurring even after thermal equilibration. This is ascribed to the high *χ*_SC_ within both aerogel types, that act as a rigid scaffold to prevent material collapse. Interestingly, the 25 wt.% PCL aerogel heats up at a slower rate than the control aerogel (with *t* < 2 min), likely owing to its intrinsic phase‐change insulation effect, with no obvious leakage of the PCL. However, at extended timeframes (i.e., *t* ∼ 2 min), the top surface temperatures of the 0 and 25 wt.% PCL aerogels converge near 75°C–90°C, which can be ascribed to the similar porosity (and by extension, thermal conductivity) of these aerogels. Moreover, regardless of aerogel composition, the minimum temperature difference between the hot plate set temperature and aerogel top surface temperature is nearly 60°C (see Figure 7C), highlighting the excellent insulation behavior of these aerogels under steady‐state heating.

To understand the role of this PCI effect on the thermal management performance of the aerogels, the aerogels were heated using a heat lamp until a heat absorption saturation transpired (i.e., reaching *T*_max_), followed by cessation of illumination. The temperature of both the aerogel's illuminated front side and non‐illuminated back was subsequently recorded, until cooled to ambient temperature (*T*_amb_ ∼ 30°C). As shown in Figure 7D, the front surfaces of both aerogels heat up rapidly (over ∼ 63 s) to a maximum temperature of ∼118°C. However, upon cessation of illumination, the aerogels rapidly dissipate the heat, reaching *T*_amb_ within about 60 s. Akin to previous heat transfer experiments illustrated in Figure 7C, the 25 wt.% PCL aerogel back surface temperature remains ∼ 6°C–7°C lower than the 0 wt.% PCL aerogel, a consequence of the PCI effect. Mimicking the radiative heat/light dissipation mechanisms characteristic in hierarchically structured plant cuticular tissues [19], aerogels fabricated via TIPS and co‐gelation exhibit excellent thermal management (i.e., heat dissipation and insulation) performance. Temperature‐dependent mechanical characterization near the PCL phase transition would provide further insight into the suitability of these materials for load‐bearing thermal management applications and represents an important direction for future work.

#### 2.4.2 Aerogel Surface Properties and Their Applications

Water‐repellency and self‐cleaning are characteristics highly sought for applications including antifouling surfaces, building insulation, and oil–water separation. Hence, developing aerogels with high water contact angles (WCA ≥  150°) is advantageous for developing multifunctional materials. Such characteristics are commonly observed on plant surfaces owing to the hierarchical surface roughness present on plant cuticular tissue (Figure 8A) that imparts hydrophobicity or superhydrophobicity, and selective permeability. From a fundamental perspective, the hydrophobicity or superhydrophobicity of plant cuticular tissue can be achieved through the Cassie–Baxter (CB) state, which prevents wetting of cuticle surfaces owing to the air‐filled hierarchical structure [83, 84].

**FIGURE 8:**
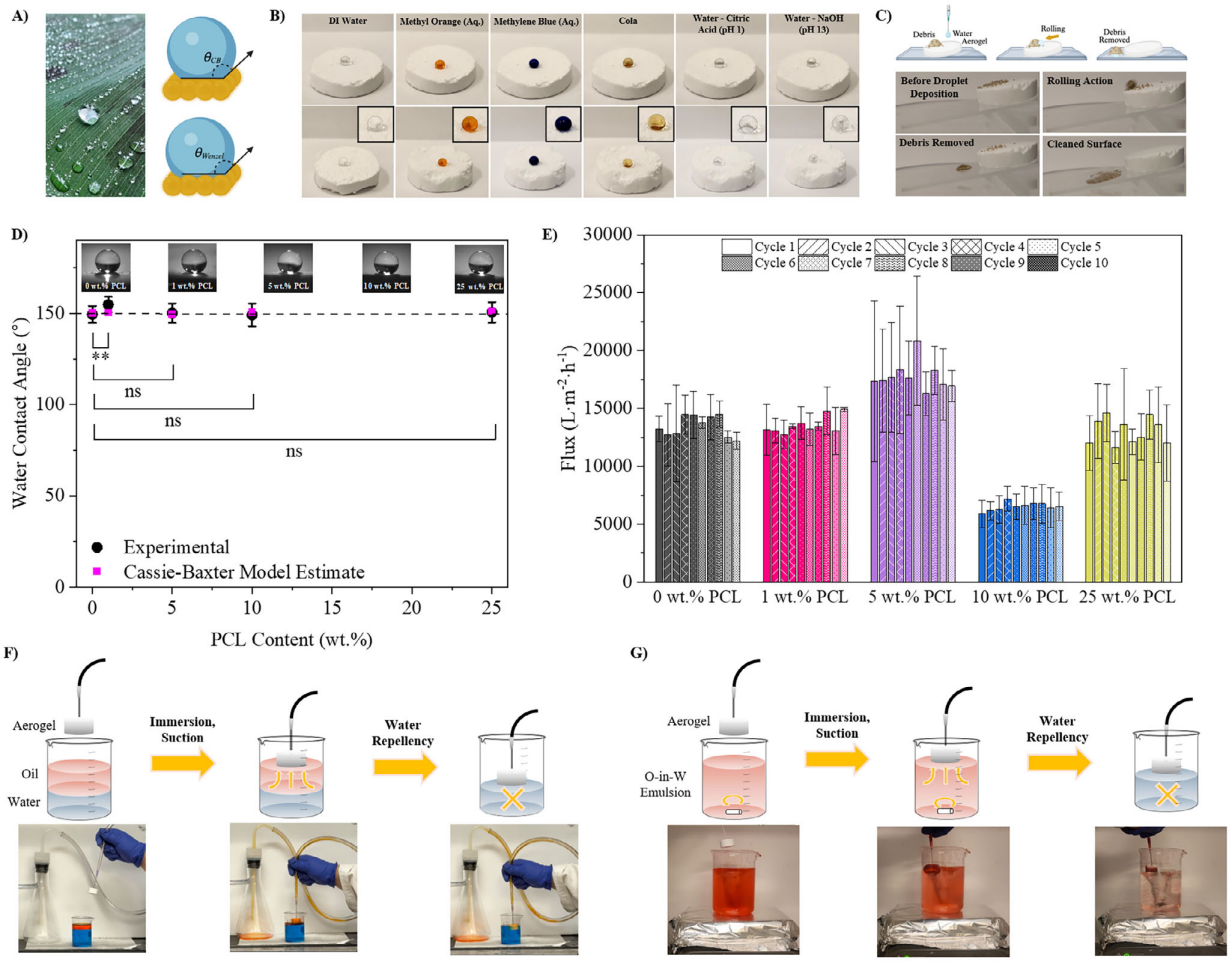
(A) Image of a banana plant leaf illustrating its highly hydrophobic surface, and schematic illustrating differences between CB and Wenzel states along the aerogel surface. (B) Droplets of various contaminant fluids deposited on the surface of a PLLA/PDLA control (top row) and 25 wt.% PCL aerogel (bottom row, inset illustrates zoomed‐in droplet for easy viewing). (C) Schematic and images illustrating the aerogel self‐cleaning mechanism with debris removed via rolling action. (D) Water contact angle of aerogels with respect to PCL content, overlaid with CB model predictions for water contact angles. (E) Continuous oil flux measurements of scCO_2_‐dried aerogels obtained over 10 sorption–desorption cycles (*n* = 3). (F) Schematic and image illustrating PLLA/PDLA control aerogel coupled to continuous oil–water separation system with the oil‐phase being hexane (top layer, dyed red with Sudan III), and aqueous‐phase (bottom layer, dyed with methylene blue hydrate) prior to immersion into the oil–water mixture, during immersion, and after complete separation of the oil‐phase from the aqueous phase. G) Schematic and image illustrating PLLA/PDLA control aerogel coupled to continuous oil–water separation system for separation of an oil‐in‐water emulsion containing hexane (oil‐phase, dyed red with Oil Red O), and water (not dyed), prior to immersion into the oil‐in‐water emulsion, during immersion, and after complete separation of the oil‐phase from the aqueous phase. Data labels of ns, *, **, ***, **** represent significance levels of *p* > 0.05, *p* < 0.05, *p* < 0.01, *p* < 0.001, and *p* < 0.0001, respectively, obtained from Dunnett's multiple comparisons test. Created in BioRender. Tuccitto, A. (2026) https://BioRender.com/yorp9x7, Created in BioRender. Tuccitto, A. (2026) https://BioRender.com/sd6o5kp.

Similar to plant cuticular tissue, this water repellency and self‐cleaning behavior is observed for the prepared aerogels, which is demonstrated in Figure 8B,C by the inability of fluid contaminants to wet the aerogel surfaces, as well as the water‐assisted self‐cleaning ability that easily removes dirt and debris from the aerogel surface. Inspired by other work [46], various fluids were deposited on the surface of aerogels containing 0 and 25 wt.% PCL, including: DI water, water dyed with methylene blue, water dyed with methyl orange, cola, an aqueous solution with pH 1, and an aqueous solution with pH 13 [49]. Here, the fluids do not wet the surface, owing to the macro‐/meso‐porous SC template imparting self‐cleaning behavior, regardless of the fluid composition or pH. Beyond this, we demonstrate the potential of our aerogels to be cleaned using simple water‐assisted self‐cleaning, by placing aerogels containing 0 or 25 wt.% PCL on a glass slide angled at < 10° (consistent with CB state self‐cleaning materials) [85] with their surfaces covered in sand, as shown in Figure 8C, and in Video S1 (0 wt.% PCL Aerogel) and Video S2 (25 wt.% Aerogel). After droplet deposition, rolling action along the aerogel surface initiates, causing the accumulation and entrapment of the debris within the rolling droplet, until the debris‐containing droplet simply rolls off, leaving a pristine surface. It should be noted that this water repellency is critical as moisture absorption can detriment the material functionality (e.g., thermal management performance) of aerogels and promote the development of fungi and mold within the porous structure [5], as is the case for plant tissues (e.g., plant cuticles that exhibit contaminant repellency to suppress blockage of sunlight).

From WCA measurements, the PLLA/PDLA control exhibited superhydrophobicity within error (WCA of 149.5° ± 4.7°) owing to the hierarchical internal structure with crystals on the order of 1–10 µm, and finer mesopores of 7–9 nm within these microstructures, as was illustrated via SEM and BET analysis. Interestingly, with 1 wt.% PCL, there is a statistically significant increase in WCA to 154.9° ± 4.3°, consistent with PCL imparting a higher *S*_BET_. Beyond this, the WCA of all other aerogels prepared are comparable to the control (150.2° ± 5.3°, 149.2° ± 6.3°, and 150.7° ± 5.6° for aerogels with 5, 10, and 25 wt.% PCL), owing to the roughness present along the undulating convex crystal structures (Figure 8D). This similar WCA among the prepared aerogels suggests that porosity (∼ 90%) is likely the most critical factor affecting experimental WCA values, with *S*_BET_ possibly resulting in WCA variations among samples.

To understand whether this superhydrophobicity can be ascribed to a CB (i.e., air entrapment between the droplet and aerogel surface) or Wenzel State (i.e., complete wetting of the aerogel surface) as is schematically illustrated in Figure 8A, a detailed description of CB contact angle model predictions can be found in the Supporting Information [83, 86], with prediction values listed in Table S8. Our experimental WCA measurements are in strong agreement with the CB Model predictions, supporting this hypothesis (Figure 8D) [83].

The superhydrophobic nature of these aerogels makes them well‐suited for applications including oil–water separation. Static sorption measurements were conducted to demonstrate the potential for sorption of nonpolar fluids, using three types of oils: a solvent (hexane), food oil (canola oil), and distillate oil (diesel) (Details found in Figure S19). From these measurements, the aerogels appear super oleophilic with nearly instantaneous sorption of the selected oils (see Supporting Information for description of Mechanistic Origin of Superhydrophobicity and Superoleophilicity). Furthermore, to demonstrate the continuous oil sorption ability of these aerogels, each aerogel was connected to a continuous oil–water separation system with needle‐coupled tubing [5, 87], which is illustrated schematically in Figure 8F. It should be noted that this testing is only possible owing to the tough mechanical performance of the prepared aerogels, which do not undergo catastrophic crack propagation upon needle insertion and during the sorption process. The pressure‐difference across the system and the oil–water mixture allows suction of low surface‐tension fluid through the aerogel. As such, we evaluate the continuous oil sorption ability of the prepared aerogels, with flux measurements displayed in Figure 8E (details found in Experimental section). With < 10 wt.% PCL, aerogels exhibited flux values ranging from 13 156–17 352 L·m^−2^·h^−1^, which decreased to as low as 5881 L·m^−2^·h^−1^ with 10 wt.% PCL. This decrease with 10 wt.% PCL coincides with the finer crystalline superstructures in this composition (Figure S6); notably, flux partially recovers at 25 wt.% PCL (12 009 L·m^−2^·h^−1^), coincident with the larger superstructure size at this loading (2.08 µm compared to 1.59 µm at 10 wt.% PCL). In fact, the observed flux values in function of PCL loading is directly mirrored in the crystalline superstructure size observed by SEM analysis (see Figure S6), with the flux value measured for each sorption–desorption cycle exhibiting a positively correlated relationship with crystalline superstructure size (Figure S20). In comparison to literature (see Table S9 for detailed comparison), the prepared aerogels perform very well, achieving flux values that are similar or greater than conventional materials used for similar oil–water separation [87, 88, 89]. The flux was evaluated over 10 cycles to demonstrate the performance stability of these materials, with no obvious significant deviations observed for each sample type.

The oil–water separation ability of the prepared aerogels is illustrated in Video S3 (images illustrated in Figure 8F), using the PLLA/PDLA control aerogel. Here, the oil‐phase was selected as hexane (top layer, dyed red with Sudan III), and aqueous‐phase (bottom layer, dyed with methylene blue hydrate). From this video, the aerogel can selectively permit the flow of hexane while actively repelling the aqueous phase. Upon removal of the aerogel from the mixture, the aqueous phase simply rolls off, while oil remaining within the aerogel can be desorbed via the applied suction pressure. To further confirm the ability of these aerogels to withstand the strong suction pressure applied during continuous oil sorption, we measure the porosity of aerogels after oil–water separation and found a negligible decrease in porosity (≅ 2.3%) which further demonstrates their exceptional mechanical robustness due to the hierarchical macro‐/meso‐porous SC template.

The oil–water separation ability of the prepared aerogels can be extended to separating oil from oil‐in‐water emulsions, as is illustrated in Video S4 (images illustrated in Figure 8G), using the PLLA/PDLA control aerogel. Here, the oil‐phase was selected as hexane (dyed red with Oil Red O). From this video, the aerogel can selectively permit the flow of hexane, even under stirring conditions, while actively repelling the aqueous phase, offering a unique application of the selective sorption characteristics of these aerogels.

#### 2.4.3 Comparison of Aerogel Structural and Bulk Property Characteristics With Literature

Finally, we compare the structural characteristics and bulk properties of the prepared aerogels with existing benchmarks including other PLA‐based aerogels and monoliths (i.e., those with and without PLA SCs), as well as typical polymer‐based aerogels and monoliths (e.g., polyimide), and a few other relevant systems, as is illustrated in Table S9. Relative to existing PLA‐based aerogels and monoliths, the aerogels in this work present the highest *S*_BET_, similar thermal conductivity, and are notably softer than existing PLA‐based aerogels. Beyond PLA‐based aerogels and monoliths, the functional properties of the aerogels in this work meet or exceed those in relevant literature. For example, the prepared aerogels offer comparable or better flux values for similar fluids, in comparison to recent literature benchmarks for other aerogels and monoliths. Beyond these characteristics, the prepared aerogels are comprised exclusively of thermoplastic blend constituents and are formed by thermoreversible gelation. As such, these aerogels can be easily re‐processed by solution or melt‐state approaches, a current limitation of state‐of‐the‐art polymer aerogels (e.g., polyimide). Solution‐state and melt re‐processing demonstrations of the 0 and 25 wt.% PCL aerogels are exemplified in Figure S21, with both aerogel types being readily dissolved in DMF, as well as being readily melt re‐processable by compression molding. Moreover, these aerogels are prepared in the absence of any other additives, and are prepared from industrially compostable materials, offering broadened end‐of‐life options for these materials, in comparison to traditional thermosetting counterparts.

## 3 Conclusion

In this work, we demonstrate that through leveraging liquid–solid de‐mixing under thermally‐induced phase separation, we fabricate aerogels comprised of exclusively spherulitic SCs with an interconnected network architecture, resembling plant cuticles. To tailor the stiffness of these aerogels, we implement polymer blending with PCL. During cooling of gels, we evidence sequential crystallization of PLA followed by PCL. This sequential crystallization approach allows the development of soft aerogels exhibiting tailorable bulk properties, without loss of functional properties. The internal structure of these gels can be preserved using scCO_2_ drying, which limits the collapse of pores relative to other drying approaches like freeze–drying. This material fabrication pathway yields hierarchically macro‐/meso‐porous aerogels that, to the best of our knowledge, present the highest internal surface areas reported in literature for SC‐based PLA aerogels (up to 186 m^2^·g^−1^). Synchrotron SAXS analysis of prepared aerogels suggests that the mesopores in these aerogels may be associated with the space between SC‐rich clusters, while the macropores are associated with the space between crystalline superstructures.

We illustrate that this internal structure resists brittle failure under compressive loading (70% Strain) and can withstand cyclic loading without significant densification (< 15% reduction over 100 cycles at 20% strain), addressing key mechanical limitations associated with the brittleness of PLA‐based aerogels. Specifically, three structural characteristics, namely the discrete spherulitic morphology, the microstructural uniformity, and the isotropic texture present in the spherulitic structure collectively circumvents edge effects that characteristically concentrate stress and initiate fracture, while imparting uniform stress distribution and facilitating energy absorption. Beyond overcoming PLA's intrinsic brittleness, aerogels prepared through this pathway exhibit great functional properties including superhydrophobicity (WCA up to 155°) ascribed to the macro‐/meso‐porous structure imparting a CB type water repellency mechanism. This gives rise to self‐cleaning ability with a wide range of aqueous media (e.g., highly acidic, basic, and dyed) being naturally repelled from the aerogel surface, in addition to dirt and contaminants being readily cleaned with water. Additionally, these aerogels are intrinsically superoleophilic and perform well in continuous oil–water separation (Flux ∼5881–17 352 L·m^−2^·h^−1^), exhibiting great structural stability during the application of suction, owing to their tough mechanical response. Interestingly, experimental flux values exhibit a positive correlation with crystalline superstructure size.

Furthermore, we achieved excellent insulation ability (*k*_Cond_ ∼ 36 mW·m^−1^·K^−1^), with PCL behaving as an interpenetrated phase‐change insulation material (i.e., leakage free) under both steady‐state and dynamic thermal treatment conditions. In addition, the SC‐based template developed imparts aerogels with fantastic geometric‐thermal stability with no obvious structural distortion during heat treatment at 150°C. This combination of properties makes these aerogels a versatile and green alternative to traditional aerogels without compromising usability, thus broadening the use of PLA‐based aerogels in formerly inaccessible applications.

To conclude, our PLA aerogels based on a bio‐inspired network‐spherulitic SC topology exhibit a unique morphological‐topological configuration that creates a strong and tough material with water repellent/self‐cleaning ability, oil–water separation capability, as well as excellent thermal insulation and geometric‐thermal shape stability. The suite of properties offered by materials prepared by this micro‐/nano‐structuring pathway are especially useful in developing compostable aerogels for lightweight, self‐cleaning insulation, and biomedical applications necessitating soft and porous structures. From the lenses of green manufacturing and structure–property relationships, this micro‐/nano‐structuring pathway provides a strategy for developing aerogels with tunable hierarchical structures and multifunctional properties, from compostable materials.

## 4 Experimental Section

### 4.1 Materials

PLLA 3001D (*M*_w_: 1.36 × 10^5 ^g·mol^−1^, *M*_n_: 7.2 × 10^4^ g·mol^−1^, 1.4% D‐isomer content, MFR: 22 g × 10 min^−1^ at 210°C with 2.16 kg) was kindly supplied from Natureworks LLC [90]. PDLA D120 (Corbion, *M*_w_: 1.5 × 10^5^ g·mol^−1^, *M*_n_: 8.9 × 10^4^ g·mol^−1^, < 1% L‐isomer content, MFR: 23 g × 10 min^−1^ at 210°C with 2.16 kg) was used [38]. PCL CAPA 6500 (*M*_w_: 5.00 × 10^4^ g·mol^−1^, MFR 7 g × 10 min^−1^ at 160 C with 2.16 kg) was supplied by Perstorp. As well, N,N‐dimethylformamide (Certified ACS, 99.8% purity) was purchased from Fisher Chemical. Anhydrous ethanol was purchased from Commercial Alcohols (Greenfield Global). Carbon dioxide (99.8% purity) was purchased from Messer Canada Inc. Hexanes (Certified ACS 98.5% purity, ϱ ∼ 0.669 g·mL^−1^) was purchased from Fisher Scientific, 100% Pure canola oil (ϱ ∼ 0.95 g·mL^−1^) was purchase from No name, and diesel (ultra‐low sulfur diesel, ULSD, ϱ ∼ 0.840 g·mL^−1^, viscosity ∼ 3.350 mPa·s) was purchased from Petro–Canada [87]. Methylene blue hydrate and Sudan III were supplied by Sigma Aldrich. Sodium hydroxide pellets (Reagent grade) were purchased from Caledon Laboratories, and anhydrous citric acid (reagent grade, purity > 99.5%) was obtained from BioShop Canada Inc. An ethyl cyanoacrylate adhesive (krazy GLUE, All purpose, KRAZY) was used in fixation of aerogels for NanoIndentation experiments.

### 4.2 Aerogel Preparation

Aerogels with varying compositions were prepared by a thermoreversible gelation process inspired by Krishnan et al. [18], using DMF as the gelation solvent, similar to our related work [91]. Briefly, 100 mL of DMF was added and sealed in a 250 mL round bottom flask and was heated to 100°C under stirring at 900 rpm. Then, PLLA, PDLA, and PCL were added in the proportions shown in Table 2, with the total polymer fraction comprising 10 wt.% of the solution. Here, commensurate fractions of PLLA and PDLA are used to maximize the fraction of SCs that comprise the aerogels. The solutions were stirred for 2 h, until the polymer fraction dissolved homogeneously. The solution was then removed from heat and rapidly dispensed into 50 mL vials in 10 mL aliquots. These aliquots were then maintained at room temperature for 30 min without stirring. Within these 30 min, the solutions became increasingly and homogeneously opaque with an increase in viscosity, indicative of liquid–solid de‐mixing (i.e., crystallization, L → C). It should be noted that with increasing PCL content in solution, the gelation time became increasingly slow. When the PCL content exceeded 25% of the polymer fraction (e.g., 50 wt.% as shown in Figures S1 and S3), obvious macroscopic phase separation occurred. Specifically, the lower layer of the mixture formed a physical gel, with the upper layer remaining liquid‐like at room temperature. However, full solidification of both layers is possible with further cooling. After the aliquots solidified, samples were stored in a freezer maintained at –22°C for 48 h to complete gelation. Gelation is complete when the solutions no longer flow when inverted. Samples were then removed from the freezer and allowed to heat to ambient temperature naturally. These gels were then subject to an annealing stage in a circulating water bath maintained at 70°C for 15 min. This annealing procedure is believed to ensure the formation of exclusively PLA SCs [18]. The samples were then removed from the water bath and cooled naturally to ambient temperature, followed by cooling at –22°C for 12 h in a freezer. The sol–gels were then removed from the freezer, allowed to heat to ambient temperature naturally, and then subject to solvent exchange to substitute the DMF from the thermogels.

**TABLE 2:** Names and compositions of fabricated aerogels.

Sample name	PLLA 3001D/PDLA D120 content (wt.% of polymer fraction)	PCL content (wt.% of polymer fraction)
0 wt.% PCL (Control)	100	0
1 wt.% PCL	99	1
5 wt.% PCL	95	5
10 wt.% PCL	90	10
25 wt.% PCL	75	25
50 wt.% PCL	50	50

*Note*: The ratio of PLLA to PDLA is fixed to be commensurate (i.e., 50/50 ratio of PLLA to PDLA) to maximize the content of SCs in aerogels.

For monoliths prepared by freeze–drying (i.e., cryogels), sol–gels were exchanged with DI water 5 times over 48 h to remove the DMF, followed by cooling in a freezer at –22°C for 12 h to stabilize the gel structure. These hydrogels were then subject to lyophilization (LABCONCO FreeZone 2.5 Plus) in a sample chamber maintained at –20°C for 3 days, yielding freeze–dried monoliths.

For monoliths prepared by scCO_2_‐drying (i.e., aerogels), ethanol was then added to the sol–gels and replaced 5 times over 48 h, to remove the DMF from the gels. Once exchanged with ethanol, the gels were removed from their respective vials and subject to exchange with liquid CO_2_ (1500 psi, 25°C) and subsequently scCO_2_ (1500 psi, 40°C), similar to related work [3]. It should be noted that these drying methods were selected as they minimize shrinkage compared to oven and vacuum‐oven drying procedures.

### 4.3 Material Characterization

Details regarding material characterization can be found in Experimental Supporting Information—Material Characterization.

### 4.4 Statistical analysis

Experimental data presented in figures are reported as mean values and standard deviations (SD)s. Statistical analyses were conducted using GraphPad Prism software. Here, One‐Way ANOVA (Analysis of Variance) was applied to the compressive modulus (Figure 6B), compressive strength (Table S7), thermal conductivity (Figure 7A), porosity (Table S1), water contact angle (Figure 8), oil sorption capacity (Figure S19), and porosity after 100 compressive cycles (20% strain), and was coupled with Dunnett's multiple comparisons test where the null hypothesis of the One‐Way ANOVA was rejected. Data labels of ns, *, **, ***, **** represent significance levels of *p* > 0.05, *p* < 0.05, *p* < 0.01, *p* < 0.001, and *p* < 0.0001, respectively. All statistical comparisons discussed are conducted between the 0 wt.% PCL control aerogel and a given PCL‐modified aerogel composition. Numerical details regarding these statistical analyses, including the number of replicates per sample for each test, can be found in Table S10.

## Author Contributions

**Anthony V. Tuccitto**: conceptualization, methodology, formal analysis, investigation, writing – original draft, and writing – review and editing. **Zeineb Ben Rejeb**: methodology, formal analysis, investigation, and writing – review and editing. **Rafaela Aguiar**: methodology, formal analysis, investigation, and writing – review and editing. **Nello D. Sansone**: methodology, formal analysis, investigation, and writing – review and editing. **Sumaiya Farzana**: methodology, formal analysis, investigation, writing – review and editing. **Isaac Ali**: methodology and formal analysis, **Jun Uk Lee**: writing – review and editing. **Daniel J. Davidson**: methodology, formal analysis, investigation writing – review and editing. **Ailsa K. Edward**: methodology, formal analysis, investigation, and writing – review and editing. **Chul B. Park**: supervision. **Patrick C. Lee**: conceptualization, writing – review and editing, funding acquisition, project administration, and supervision.

## Conflicts of Interest

The authors declare no conflicts of interest.

## Supporting information



Experimental details, images of prepared aerogels, images of 50 wt.% PCL thermogels, flow‐chart illustrating possible gelation pathways in polymer blends, optical microscope image illustrating thermogel structure, calorimetric 1st heating thermograms for select thermogels, envelope density and porosity of blend aerogels, violin plot and histograms showing size distributions corresponding to microstructural SEM images, SEM images of freeze–dried PLLA/PDLA monoliths, calorimetric thermograms of aerogels (varying heating rate, zoomed in SC endotherms, modulation temperature profile), graphical illustration of TMDSC principles, explanation of TMDSC principles, extracted calorimetric data, TGA derivative weight loss, TGA analysis of neat aerogel constituents, nanoindentation topography and gradient images, TGA weight loss percentages, full FTIR spectra obtained for scCO_2_‐dried aerogels, FTIR spectral assignment, 2D correlation description/analysis, SAXS T‐S Fitting parameters, compressive stress–strain curve of freeze–dried 0 wt.% PCL aerogel, Ashby plot of envelope density with respect to modulus for biopolymer aerogels, envelope density and density change after compressive testing, thermal insulation and management testing apparatus schematic, infrared thermal camera images from thermal insulation and thermal management testing, CB model description, comparison between experimental and predicted WCA measurements, static oil sorption capacity measurements, relationship between flux and crystalline superstructure size over 10 sorption–desorption cycles, statistical analysis details, and demonstration of aerogel recyclability and re‐processability (.docx).
**Supporting File 1**: adma75136‐sup‐0001‐SuppMat.docx.

**Supporting File 2**: adma75136‐sup‐0002‐VideoS1.mp4.

**Supporting File 3**: adma75136‐sup‐0003‐VideoS2.mp4.

**Supporting File 4**: adma75136‐sup‐0004‐VideoS3.mov.

**Supporting File 5**: adma75136‐sup‐0005‐VideoS4.mp4.

## Data Availability

The data that supports the findings of this study are available in the Supporting Information of this article. Additional supporting data are available upon request.
