# Supplementary material for: A Thermoreversible Gelation Pathway to Plant Cuticle‐Inspired Biopolymer Aerogel Monoliths Based on Network‐Spherulitic Crystal Assemblies
Source: Adv Mater. 2026 Sep 29;38(63):e75136. doi: 10.1002/adma.75136 (PMC13755654; doi:10.1002/adma.75136)
Supplement: Supplementary file 1 [file ADMA-38-e75136-s001.docx]

­­Supporting Information

A Thermoreversible Gelation Pathway to Plant Cuticle-Inspired Biopolymer Aerogel Monoliths based on Network-Spherulitic Crystal Assemblies

Anthony V. Tuccitto ^a,b^, Zeineb Ben Rejeb ^b^, Rafaela Aguiar ^a^, Nello D. Sansone ^a^, Sumaiya Farzana ^a^, Isaac Ali ^a^, Jun Uk Lee ^a^, Daniel J. Davidson ^c^, Ailsa K. Edward ^d^, Chul B. Park ^b *^, Patrick C. Lee ^a *^

*Corresponding Authors: [patrick.c.lee@utoronto.ca](mailto:patrick.c.lee@utoronto.ca), [park@mie.utoronto.ca](mailto:park@mie.utoronto.ca)

^a^Multifunctional Composites Manufacturing Laboratory (MCML), Department of Mechanical and Industrial Engineering, University of Toronto, 5 King’s College Road, Toronto, M5S 3G8, Canada.

^b^Microcellular Plastics Manufacturing Laboratory (MPML), Department of Mechanical and Industrial Engineering, University of Toronto, 5 King’s College Road, Toronto, M5S 3G8, Canada.

^c^ Department of Chemical Engineering and Applied Chemistry, University of Toronto, Toronto, Ontario, M5S 3B3, Canada.

^d^ Department of Chemistry, University of Toronto, Lash Miller Chemical Laboratories, 80 St. George Street, Toronto, M5S 3H6, Canada.

Table of Contents

[Experimental Supporting Information – Material Characterization	3](#_Toc238223064)

[*Calorimetric analysis of thermogels*	3](#_Toc238223065)

[*Polarized optical microscopy*:	3](#_Toc238223066)

[*Scanning electron microscopy*	3](#_Toc238223067)

[*Nitrogen adsorption-desorption isotherms*	3](#_Toc238223068)

[*Calorimetric analysis of monoliths*	3](#_Toc238223069)

[*Nanoindentation*	4](#_Toc238223070)

[*Fourier-transform infrared spectroscopy (FTIR)*	5](#_Toc238223071)

[*Synchrotron* Small Angle X-Ray Scattering *(SAXS)*	5](#_Toc238223072)

[*Compression tests*	5](#_Toc238223073)

[*Water contact angle measurements*	6](#_Toc238223074)

[*Static oil sorption capacity*	6](#_Toc238223075)

[*Continuous oil flux performance*	6](#_Toc238223076)

[*Thermal conductivity*	6](#_Toc238223077)

[*Thermogravimetric analysis*	7](#_Toc238223078)

[*Infrared camera imaging*	7](#_Toc238223079)

[*Recycling/reprocessing of aerogel monoliths*	7](#_Toc238223080)

[Material Preparation – Supporting Information	8](#_Toc238223081)

[Structural and Bulk Property Characterization – Supporting Information	10](#_Toc238223082)

[Thermal Analysis – Supporting Information	12](#_Toc238223083)

[Nanoindentation Analysis – Supporting Information	15](#_Toc238223084)

[FTIR Analysis – Supporting Information	16](#_Toc238223085)

[Synchrotron Small Angle X-ray Scattering Analysis – Supporting Information	21](#_Toc238223086)

[Mechanical Property – Supporting Information	22](#_Toc238223087)

[Thermal Insulation and Thermal Management Assessment – Supporting Information	24](#_Toc238223088)

[Cassie-Baxter Model Predictions– Supporting Information	25](#_Toc238223089)

[Mechanistic Origin of Superhydrophobicity and Superoleophilicity	26](#_Toc238223090)

[Static Sorption Capacity – Supporting Information	27](#_Toc238223091)

[Relationship between Flux and Crystalline Superstructure Size – Supporting Information	28](#_Toc238223092)

[Comparison with State-of-the-Art	29](#_Toc238223093)

[Demonstration of Material Re-processing	31](#_Toc238223094)

[Statistical Analysis – Quantitative Evidence	32](#_Toc238223095)

[References	34](#_Toc238223096)

# **Experimental Supporting Information – Material Characterization**

*Calorimetric analysis of thermogels*: A TA Instruments DSC 250 was used to characterize the phase change behaviour of select thermogels. Approximately 5 – 10 mg of a given thermogel (treated at −22 °C, prior to annealing) was sealed in an aluminum hermetic pan, and was subject to heating to 135 °C at a rate of 10 °C×min^-1^ under *N_2_* gas flow. Samples were then held isothermally for 5 min, and were subsequently cooled at 20 °C×min^-1^ to −40 °C. Samples were then held isothermally for 2 minutes, followed by re-heating at a rate of 10 °C×min^-1^.

*Polarized optical microscopy*: Polarized optical microscopy (POM) was used to visualize the crystalline structure of the control thermogel visually. Here, a small aliquot of freshly prepared thermogel (i.e., before cooling in a freezer for further gel consolidation) was sandwiched between two glass slides and spread until thin enough until it appeared nearly transparent. Images were taken using a Polarized optical microscope (BX53, Olympus, Tokyo, Japan), equipped with a 50× objective lens and an Olympus U-TP530 wave plate.

*Density and porosity*: The envelope density of the aerogels was determined from their mass-to-volume ratio. The porosity of the aerogels was quantified using the following equation:

$$Porosity=\left[ 1-\frac{\rho_{\mathrm{aerogel}}}{\rho_{\mathrm{polymer}}} \right]\times100\%$$

(S1)

Here, the $\rho_{\mathrm{aerogel}}$ is the mass/volume ratio of the aerogel, while the $\rho_{\mathrm{polymer}}$ is a weighted average of the polymer blend component’s bulk density ($\rho_{\mathrm{PLLA}}$ = 1.24 g×cm^-3^, $\rho_{\mathrm{PDLA}}$= 1.24 g×cm^-3^, $\rho_{\mathrm{PCL}}$= 1.1 g×cm^-3^).

*Scanning electron microscopy*: A FEI Quanta FEG 250 Scanning electron microscope was used for morphological characterization of the fabricated monoliths. Cross-sections of each aerogel were cut with a razor blade, and were sputter coated with a layer of carbon (Carbon evaporator EMITECH K950X). It should be noted that the biopolymers used (especially PCL) are prone to degradation under the electron beam. As such, specimens were imaged under an acceleration voltage of 1 kV. Crystal size distributions were obtained from SEM micrographs captured at 5,000, 20,000, 50,000, and 100,000× magnification, averaging 50 measured crystal diameters, with corresponding standard deviation values reported.

*Nitrogen adsorption-desorption isotherms*: Nitrogen adsorption-desorption isotherm characterization was conducted using an autosorb iQ automated gas analyzer (Quantachrome Instruments) as described in related work.^[1]^ Each aerogel was degassed under vacuum at 30 °C prior to measurements. The Brunauer-Emmett-Teller (BET) method was applied to quantify the specific surface area (*S_BET_*) of the aerogels at relative pressures < 0.3. The calculation model used was *N_2_* at 77K on carbon (slit pore, NLDFT equilibrium model) based on the shape of the adsorption-desorption isotherms.

*Calorimetric analysis of monoliths*: A TA Instruments DSC 250 was used to characterize the crystal structure of the polymers comprising the monoliths. Specifically, aerogel specimens were cut to a mass of 5 – 10 mg, sealed in an aluminum hermetic pan, and were heated at a rate of 10 °C×min^-1^ under N_2_ gas flow. The DSC was calibrated with indium prior to analysis. All calorimetric analysis was conducted in triplicate to validate repeatability and sample consistency, with representative thermograms reported.

The degree of crystallinity of SCs ($\chi_{\mathrm{SC}}$) were quantified using the following equation.

$$\chi_{\mathrm{SC}}=\frac{\Delta H_{m,SC}}{\Delta H_{m,SC}^{0}\cdot w_{\mathrm{PLA}}}\times100\%$$

(S2)

where *ΔH_m,SC_* is the melting enthalpy of the SC endotherm obtained by calorimetric analysis, *ΔH^0^_m,SC_* is the theoretical enthalpy of fusion for 100 % SCs (142 J×g^-1^),^[2]^ and *w_PLA_* is the fraction of PLA in the monolith (i.e., the sum of the PLLA and PDLA fraction).

The degree of crystallinity of PCL ($\chi_{\mathrm{PCL}}$) was estimated using the following equation:

$$\chi_{PCL}=\frac{\Delta H_{m,PCL}}{\Delta H_{m,PCL}^{0}\cdot w_{\mathrm{PCL}}}\times100\%$$

(S3)

Where *ΔH_m,PCL_* is the melting enthalpy of the PCL endotherm obtained by calorimetric analysis, *ΔH^0^_m,PCL_* is the theoretical enthalpy of fusion for 100% PCL crystals (135 J×g^-1^),^[3][4]^ and *w_PCL_* is the fraction of PCL in the monolith.

Modulated differential scanning calorimetry (TM-DSC) was conducted on select aerogel specimens, to deconvolute overlapping thermal phase transitions present in the aerogels. TM-DSC applies a non-linear heating profile to decompose the heat flow signal from a given material during a given thermal phase transition, as either reversing, or non-reversing heat flow signals. First, 5 – 10 mg of an aerogel specimen was carefully sealed in an aluminum hermetic pan. The applied temperature modulation procedure was conducted in accordance with recommendation from TA Instruments.^[5]^ First, the sample was equilibrated at –80 °C and treated with an underlying heating rate of 0.5 °C×min^-1^ to a maximum temperature of 260 °C, using a modulation period of 100 sec and an amplitude of 1 °C. These parameters permit at least 6 full modulation cycles within the phase transitions spanning the smallest temperature range (i.e., *T_g_* of PLA), to enable full resolution of such thermal phase transitions. TM-DSC data was shifted by an arbitrary value for viewing clarity.

*Nanoindentation*: Aerogels containing either 0 or 5 wt.% PCL were first fixed onto a flat stub using a cyanoacrylate-based adhesive and were impregnated with the adhesive to ensure an adequately flat surface for nanoindentation analysis (i.e., to fill the pores of the aerogel so they would not interfere with scanning probe microscopy (SPM) imaging). After application, the adhesive was allowed to dry for 24 h under room temperature conditions, after which the material was cryofractured to expose a representative cross-section. To establish the stiffness contribution of the adhesive, a control sample consisting of cyanoacrylate glue alone was also deposited on the stub and indented under the same conditions (as shown in **Figure S12**). Nanoindentation was performed using a Hysitron TS 77 Select Nanoindenter (Bruker, Massachusetts, United States) equipped with a standard Berkovich tip, with four replicate aerogel samples tested for each composition. Topography and gradient images were acquired with a scanning size of 5 × 5 µm, scanning rate of 0.5 Hz, a set point force of 5 µm, and a scan resolution of 256 × 256 pixels in SPM mode. Using the identified regions, arrays of indents (5 × 5) were applied with a spacing of 1 µm, employing a load profile that involved indenting to 800 µN, holding the peak load, and subsequently unloading in a load-controlled serpentine piezo-translation protocol. Pre-indentation imaging, combined with controlled spacing and positioning of the indents, allowed preferential selection of indentations centered on the spherulitic structures. Positioning the indents in this manner minimized boundary effects, and the *E_r_* was then calculated by averaging the indentation values obtained for each spherulite. The measured *E_r_* of the adhesive (2.39 ± 0.05 GPa) was substantially lower than that of the spherulitic structures in the aerogels, enabling clear distinction of the respective phases in the nanoindentation results.

*Fourier-transform infrared spectroscopy (FTIR)*: FTIR spectroscopy was conducted directly on aerogel specimens to distinguish whether physical or covalent bonding interactions are responsible for the observed aerogel morphology and bulk properties. Briefly, a Thermo Scientific iS50 ATR FTIR spectrometer was used with 128 scans and 4 cm^-1^ resolution. Moreover, FTIR spectroscopy was used to validate the DSC measurements and confirm the presence of the SC crystal polymorph as has been done in previous work.^[6]^

*Synchrotron* *small angle x-ray scattering (SAXS):* Small Angle X-ray scattering was conducted directly on aerogel specimens to understand the structural configuration of aerogel constituents. SAXS experiments were performed at the Canadian Light Source (CLS) synchrotron using the Brockhouse X-ray Diffraction and Scattering (BXDS) sector Wiggler Low Energy (WLE) beamline.^[7]^ The photon energy was 9871.3 eV selected using a Si(111) monochromator. Diffraction patterns were collected with a Rayonix MX300 CCD detector (300 mm × 300 mm active area with 73.242 μm × 73.242 µm pixels). The sample to detector distance was 2337 mm using a 4.0 mm diameter beamstop. The data were calibrated using silver behenate standard and reduced to 1D plots using GSAS-II version 5789.^[8]^ SAXS data were further processed using BioXTAS RAW 2.^[9]^

$$I\left( q \right)=\frac{A}{a+c_{1}q^{2}+c_{2}q^{4}}$$

(S4)

$$d=2\pi\left[ {\frac{1}{2}\left( \frac{a}{c_{2}} \right)}^{0.5}-\frac{1}{4}\left( \frac{c_{1}}{c_{2}} \right) \right]^{-0.5}$$

(S5)

Here, I(q) represents the scattering intensity, *q* represents the scattering vector, d represents the average SC inter-cluster distance, while A, a, c_1_, and c_2_ represent parameters obtained by nonlinear least-squares fitting of SAXS data in the correlation peak region.

*Compression tests*: Compression tests were conducted using an Instron Universal Testing System (Model 5965) equipped with a compression fixture. Details of the experimental setup can be found elsewhere.^[10]^ First, compression tests were conducted on aerogels at a crosshead speed of 1 mm×min^-1^, to a maximum strain of 70 %, after applying a pre-loading force of 0.8 N to ensure the sample surface was fully in contact with the compression fixture, to understand the failure mode of the prepared materials.

Cyclic compression tests were also conducted on aerogels at a crosshead speed of 1 mm×min^-1^, at 20 % strain, over 100 cycles, after applying a pre-loading force of 0.8 N to ensure the sample surface was fully in contact with the compression fixture. A total of 100 cycles was selected for mechanical testing for the sake of comparison with related literature.^[11]^ The compressive modulus was measured from the slope of the linear elastic region prior to the aerogel’s densification. The diameter and height of aerogels prior to compression testing was measured and recorded prior to testing for normalization of stress-strain behavior, being approximately 24 mm in diameter and 8 mm in height for each sample. The density of aerogels was recorded after cyclic compression testing to validate the extent to which densification occurred during compression.

*Water contact angle measurements*: Water contact angle (WCA) measurements were conducted using an in-house Goniometer. Images were obtained using a Pulnix Dual Tap AccuPiXEL camera, with a water droplet of approximately 2 µL deposited onto each aerogel’s surface. WCAs were obtained from images via processing using ImageJ software, with measured values averaged and reported with standard deviations (see **Table S8** for number of replicate measurements).

*Static oil sorption capacity*: The static oil-sorption capacity of the prepared aerogels was measured by immersing the aerogels in 10 mL of an oil phase for 10 min. Aerogel mass was measured before and after immersion, with the static oil sorption capacity determined as follows, as was conducted in related work.^[1]^

$$Q=\frac{m_{f}-m_{0}}{m_{0}}$$

(S6)

Here, *Q* is the static oil sorption capacity in g of oil per gram of aerogel, m_0_ is the initial mass of the aerogel prior to immersion, and m_f_ is the final mass of the aerogel after immersion. The oil phases used include hexane, canola oil, and diesel. Each static oil sorption measurement was conducted using n = 3 or n = 4 (see **Table S10**), with average values and standard deviations reported.

*Continuous oil flux performance*: To evaluate the continuous oil flux performance of prepared monoliths, aerogels were coupled to a vacuum pump, for continuous and simultaneous oil sorption and desorption. Here, a needle (BD Blunt Fill Needle, 18G × 1½ TW, 1.2mm × 40 mm) was inserted into the aerogel, connecting the aerogel to the pump. The aerogel’s bottom surface was maintained at the surface of the 10 mL aliquot of Hexane. The aerogels were subject to vacuum (65 cmHg), as has been conducted in related work.^[1]^ The oil flux (F) was calculated as follows.

$$F=\frac{V}{A\cdot t}$$

(S7)

Here, *V* is the volume of oil passing through the aerogel over time (*t*) via flow through the aerogel area (*A*) in contact with the hexane. The continuous oil-water separation process was also evaluated under identical conditions, except with water beneath the oil-phase. Upon full sorption/desorption of the oil phase, the aerogel remains in contact with the water phase, thereby validating its oil-water separation performance. It should be noted that the flux values in this work, and in other related work in literature, are highly dependent on system-related parameters (i.e., pump type, applied vacuum pressure, pressure drop across the needle, etc.).

A demonstration of how the oil-water separation ability of the prepared aerogels can be used to separate oily phases from water in non-stabilized emulsions under stirring was also conducted. Here, 250 mL of DI water was added to a 400 mL Pyrex beaker with a stir bar. To the water was added 25 mL of hexanes dyed with oil red O, under stirring at 900 rpm. This was found to be an effective stir speed to create an oil-in-water emulsion. After 10 mins of stirring, the aerogel (0 wt.% PCL) was then coupled to the pump and immersed. The oil-water separation process was recorded until no visible changes in color of the solution were detected.

*Thermal conductivity*: Thermal conductivity measurements were conducted on fabricated aerogels using a Hot Disk TPS2500S thermal constants analyzer from Hot Disk Instruments. A 7577F1 Kapton-insulated sensor with diameter of 2.00 mm was used for measurements, with n = 5 or n = 6 (see **Table S10**).

*Thermogravimetric analysis*: Thermogravimetric analysis (TGA) was conducted using a TA Instruments TGA Q50 to understand the decomposition behaviour and thermal stability of prepared aerogels. Approximately 5 – 10 mg of each respective aerogel was placed in a platinum pan and was heated from 25 to 800 °C at a rate of 10 °C×min^-1^, under nitrogen flow of 20 mL×min^-1^.

*Infrared camera imaging*: Infrared camera imaging was conducted to demonstrate the insulating ability of the fabricated aerogels, with the experimental setup illustrated in **Figure S16**. Specifically, aerogels containing 0 and 25 wt.% PCL with a thickness of ~5 mm were placed on a hot plate (Thermoscientific CIMAREC+) with a set temperature of 150 °C. The aerogel was encircled with a cork ring to prevent rapid heat loss to the environment. Thermal camera images were obtained from above the aerogel (i.e., a top-down view) using a HT-18 Thermal Imaging Camera (working temperature range of –20 – 300 °C, 22 × 160 resolution). Images were obtained before placing the aerogel on the hot plate, immediately upon placing the aerogel on the hot plate, and every 30 seconds for a duration of 2 min, and every minute thereafter, until each aerogel’s top surface temperature plateaued (10 min). Prior to every heating treatment, each aerogel composition was placed in a freezer maintained at −13 °C for 10 min to ensure the PCL be crystallized at the start of the test.

The thermal management behavior of the prepared aerogels were also evaluated using a method discussed in detail in our recent publications.^[12][13]^ Here, the front surface of aerogels (5 mm thickness) containing either 0 or 25 wt.% PCL were heated using a SCHOTT light source (intensity of 140 klx) at a fixed distance, until heat absorption saturation was achieved (~ 118 °C for both sample types). Specimens were cooled to *T_amb_* (30 °C) with the cessation of illumination, while simultaneously measuring both the front and back surface temperatures of the aerogel using an Infrared Thermal camera (TOPDON, Model: TC002). Prior to every heating cycle, each aerogel composition was also placed in a freezer maintained at −13 °C for 10 min to ensure the PCL be crystallized. It should be noted that *t* = 0 represents the instance the light is turned on.

*Recycling/reprocessing of aerogel monoliths*: To illustrate the recyclability/re-processability of prepared monoliths, 0 and 25 wt.% PCL were subjected to either solution or melt reprocessing. Prior to both reprocessing approaches, aerogels were cut into a coarse powder and were dried under vacuum at 60 °C for 6 h to remove any bound moisture. For solvent processing, dried powders were dissolved in ~ 4 mL of DMF (concentration of 10 wt./v) heated to 100 °C on a hot plate under stirring. The prepared aerogels fully dissolved in DMF within 30 min. Once homogenized, the solutions were simply removed from heat and allowed to gel. For 0 wt.% PCL, gelation transpired within ~13 minutes at ambient temperature. For 25 wt.% PCL, gelation transpired slowly at ambient temperature, and was accelerated by freezing at −13 °C for the sake of demonstration. For melt reprocessing, 0 and 25 wt.% PCL, was sandwiched between two Kapton sheets, melted on a Carver Hydraulic press for 4 min, and subjected to compression at 41.36 MPa (6000 psi) at 210 ºC. It should be noted that 0 wt.% PCL was subjected to compression at 230°C, while 25 wt.% PCL was subjected to heating at 210°C, owing to the lower viscosity of the PCL-containing aerogel. This process produced a transparent film of each blend type.

# **Material Preparation – Supporting Information**

**Figure S1:** Images of select aerogels containing 0, 25, and 50 wt.% PCL obtained from scCO_2_-drying. The 50 wt.% PCL aerogel exhibited macroscopic phase separation resulting in a gradient-structured aerogel.

**Figure S2:** Calorimetric heating thermograms obtained for thermogels with varying PCL loading, treated at –22 °C, prior to annealing, using a heating rate of 10 ^⚬^C×min^-1^. Calorimetric thermograms are shifted by an arbitrary value for viewing clarity.

**Figure S3: (Left)** Image of thermogel containing 50 wt.% PCL after cooling from 100 ^⚬^C to T_amb_ (i.e., after gelation of PLA, before gelation of PCL), **(Right)** Image of thermogel containing 50 wt.% PCL after gel consolidation in the freezer (i.e., after gelation of both PLA and PCL).

**Figure S4:** Flow-chart illustrating some possible pathways of thermally induced phase separation in single-polymer and multi-polymer systems.

**Figure S5:** Optical microscope image illustrating the network-spherulitic structure of the 0 wt.% PCL (Control) thermogel formed immediately after blending and cooling to ambient temperature (50 × objective lens). This thermogel represents a system where the network morphology/topology is formed from a Multi-Polymer System (Co-gelation of PLLA and PDLA) wherein concurrent gelation transpires via L$\to$C de-mixing, in a cooperative manner, with PLLA and PDLA co-crystallizing into a single crystal structure (i.e., stereocomplex crystal structure).

# **Structural and Bulk Property Characterization – Supporting Information**

**Table S1:** Density and porosity of prepared aerogels. All values are reported as averages from triplicate measurements (n = 3).

| Sample Name | Envelope Density (gᐧcm^-3^) | Porosity (%) |
| --- | --- | --- |
| PLLA/PDLA | 0.132 ± 0.006 | 89.4 ± 0.5 |
| 1 wt.% PCL | 0.130 ± 0.003 | 89.5 ± 0.3 |
| 5 wt.% PCL | 0.137 ± 0.002 | 88.9 ± 0.2 |
| 10 wt.% PCL | 0.126 ± 0.003 | 89.8 ± 0.2 |
| 25 wt.% PCL | 0.121 ± 0.006 | 89.9 ± 0.5 |
| 50 wt.% PCL | 0.148 ± 0.012 | 88.1 ± 1.5 |

**Figure S6: A)** Violin plot illustrating the size distribution of the spherulitic superstructures imaged in aerogels via scanning electron microscopy analysis. Histogram illustrating the size distributions of crystals, including **B)** 0 wt.% PCL, **C)** 1 wt.% PCL, **D)** 5 wt.% PCL, **E)** 10 wt.% PCL, and **F)** 25 wt.% PCL, reported with means and number of crystals counted.

**Figure S7:** Scanning electron micrograph obtained from freeze-dried PLLA/PDLA monoliths obtained at **A)** 100,000, **B)** 50,000, **C)** 20,000, and **D)** 5,000 × magnification.

# **Thermal Analysis – Supporting Information**

**Table S2:** Thermal properties of scCO_2_-dried aerogels obtained via calorimetric heating profiles, using a heating rate of 10 °C×min^-1^.

| **Sample Name** | ***T_g,PLA_* (°C) ^a^** | ***T_m,PCL_* (°C)** | ***∆H_m,PCL_* (J**ᐧ**g^-1^)** | ***χ_c,PCL_* (%)^c^** | ***T_m,SC_* (°C) ^b^** | ***∆H_m,SC_***  **(J**ᐧ**g^-1^)** | ***χ_c, SC_* (%)** |
| --- | --- | --- | --- | --- | --- | --- | --- |
| **PLLA/PDLA** | 60.1 | - | - | - | 221.7 | 87.2 | 59.7 |
| **1 wt.% PCL** | - | - | - | - | 220.8 | 84.7 | 60.3 |
| **5 wt.% PCL** | - | ≈ 48.8 | ≈ 0.278 | ≈ 4.12 | 221.7 | 83.0 | 61.5 |
| **10 wt.% PCL** | - | ≈ 39.7 | ≈ 1.59 | ≈ 11.8 | 223.2 | 79.2 | 61.9 |
| **25 wt.% PCL** | - | 48.0 | 11.8 | ≈ 35.0 | 223.2 | 66.6 | 62.6 |

^a^*T_g,PLA_* is not reported for aerogels containing PCL owing to convolution of *T_m,PCL_* with *T_g_* of PLA, as well as owing to the high degree of crystallinity of PLA that lessens the magnitude of *T_g,PLA_*.

^b^All *T_m_* values are reported as peak values.

^c^Approximate values of crystallinity are reported for PCL owing to convolution of *T_m,PCL_* with *T_g_* of PLA.

**Figure S8:** DSC heating thermogram illustrating magnified SC melting endotherm, Calorimetric thermograms are shifted by an arbitrary value for viewing clarity.

**Figure S9: A)** DSC heating thermograms obtained for the 25 wt.% PCL aerogel using heating rates ranging from 5 – 40 °C×min^-1^. Calorimetric thermograms are shifted by an arbitrary value for viewing clarity.

**Underlying Principles and Analysis of TM-DSC**

By applying an oscillatory heating-cooling profile with an underlying heating ramp, at a fixed amplitude, it is possible to decompose the heat flow signal into reversing and non-reversing heat flow signals. While all phase changes are observable (and may be convoluted) in the total heat flow signal, the reversing heat flow is indicative of reversible thermal processes including glass transition/vitrification, the endothermic portion of melting,^[14–17]^ while non-reversing heat flow is often indicative of cold crystallization, evaporation, melting, re-crystallization, or crystal reorganization.^[14–17]^

**Figure S10:** TMDSC thermogram obtained for 25 wt.% PCL Aerogel overlaid with the applied sinusoidal temperature modulation, illustrating > 6 cycles within the convoluted phase-change region. Calorimetric thermograms are shifted by an arbitrary value for viewing clarity.

From the reversing heat flow signal, devitrification of PLA (*T_g,PLA_*) is observable near 50.8 °C, which is successfully deconvoluted from the obvious endotherm near 52.6 °C in the non-reversing heat flow curve corresponding to *T_m,PCL_*. It should be noted that the magnitude of PLA’s *T_g_* is quite small owing to PLA’s high degree of crystallinity. Given the small contribution of PLA’s *T_g_* to the total heat flow signal, we believe crystallinity estimates in **Table S2** to be reliable. Beyond this, an endotherm is observable in the reversing heat flow curve near 213.2 °C corresponding to the fusion of PLA SCs. Interestingly, there is evidence of re-crystallization of PLA SCs observable with a subtle exotherm found in the non-reversing heat flow curve concurrently with the fusion observed in the reversing heat flow curve. A similar phenomenon has been observed and has been ascribed to the melting and subsequent re-organization of the less-ordered PLA crystal domains^[18]^ akin to template-effects described elsewhere,^[19]^ with the more predominant proportion of higher-order SCs subsequently melted. Finally, upon further heating, the higher-order SCs melt near 224.3 °C, as is evidenced in the non-reversing and total heat flow curves.

**Figure S11: A)** Derivative weight loss with respect to temperature is provided for scCO_2_-dried aerogels, **B)** weight loss with respect to temperature for neat materials used in aerogel preparation.

**Table S3:** Weight loss percentage at various temperatures obtained via thermogravimetric analysis for scCO_2_-dried aerogels.

| Sample Name | T_5%_ (°C) | T_50%_ (°C) | T_75%_ (°C) | T_90%_ (°C) |
| --- | --- | --- | --- | --- |
| PLLA 3001D | 329.7 | 356.4 | 371.6 | 379.6 |
| PDLA D120 | 334.0 | 351.1 | 367.9 | 376.7 |
| PCL CAPA 6500 | 336.2 | 392.4 | 403.7 | 414.7 |
| 0 wt.% PCL | 333.5 | 365.0 | 374.0 | 380.4 |
| 1 wt.% PCL | 333.7 | 366.9 | 376.1 | 382.5 |
| 5 wt.% PCL | 337.1 | 369.4 | 378.4 | 385.3 |
| 10 wt.% PCL | 336.0 | 368.4 | 377.5 | 385.7 |
| 25 wt.% PCL | 334.9 | 370.3 | 383.3 | 404.8 |

# **Nanoindentation Analysis – Supporting Information**

**Figure S12:** Representative SPM images illustrating topography for the **A)** 0 wt.% PCL aerogel, **B)** 5 wt.% PCL aerogel, and **C)** cyanoacrylate fixation adhesive. Nanoindentation gradient for the **D)** 0 wt.% PCL aerogel, **E)** 5 wt.% PCL aerogel, and **F)** cyanoacrylate fixation adhesive. All nanoindentation analyses were conducted on aerogels infiltrated with the cyanoacrylate fixation adhesive.

The reduced modulus (*E_r_*), is a material property used to describe the stiffness of a material under localized deformation such as indentation or contact loading, and accounts for both the intrinsic stiffness of the material and the influence of the contacting probe–material interface.^[20][21]^ The *E_r_* of the spherulitic structure was determined by averaging the values within the detectable boundary of each phase, enabling direct comparison across the aerogels.

# **FTIR Analysis – Supporting Information**

**Figure S13:** FTIR spectra obtained for scCO_2_-dried aerogels over full-spectral range.

**Table S4:** Assignment of FTIR spectral bands illustrating wavenumber and deformation mode, of each respective material, as well as their respective functional groups, chain conformers, or helical structure.

| Wavenumber(cm^-1^) | Deformation Mode | Material | Corresponding Functional Group/ Chain Conformer/ Helical Structure | Spectral Band |
| --- | --- | --- | --- | --- |
| 3482 | Overtone (Stretching) | PLA | C=O^[22]^ | 2 *v*(C=O)_Overtone,PLA_ |
| 2993 | Asymmetric Stretching | PLA | Asymmetric CH_3_ stretching of PLA ^[23][24][25][26]^, C–H stretching^[27]^ of methyl stretching (PLA), hydrogen bonded^[28]^ | *v*(C−H)_As,PLA_ |
| 2879 | Stretching | PLA | C_α_−H (i.e., methine)^[24][26][29]^ | *v*(C_α_-H)_PLA_ |
| 2943 | Symmetric Stretching | PLA | CH_3_^[23][26][27]^ | *v*(C–H)_S,PLA_ |
| 2943 | Asymmetric Stretching | PCL | Asymmetric C-H stretching,^[30]^ crystalline methylene next to ester’s oxygen atom^[31][32]^ | *v*(C−H)_As,PCL_ |
| 2895 | Stretching | PCL | Methylene group next to ester carbonyl ^[31]^ | *v*(CH_2_)_,PCL_ |
| 2877 - 2866 | Symmetric Stretching | PCL | CH_2_^[30][32]^ | *v*(C−H)_S,PCL_ |
| 1777 | Stretching | PLA | *gg* chain conformer, amorphous/interphase^[33][34]^ | *v*(C=O)*_gg_*_,PLA_ |
| 1768 | Stretching | PLA | *tg* chain conformer, amorphous/interphase^[33][34]^ | *v*(C=O)*_tg_*_,PLA_ |
| 1759 | Stretching | PLA | *gt* chain conformer^[33][34]^ | *v*(C=O)*_gt_*_,PLA_ |
| 1749 | Stretching | PLA | *tt* chain conformer^[33][34]^ | *v*(C=O)*_tt_*_,PLA_ |
| 1745 | Symmetric Stretching | PLA | *tt*-conformation of 3_1_ SC Helix | *v*(C=O)_S,PLA_ |
| 1735 | Stretching | PCL | C=O, amorphous^[35][36]^ | *v*(C=O) _PCL_ |
| 1727 - 1729 | Stretching | PCL | Free C=O (i.e., non-hydrogen bonded)^[37]^ | *v*(C=O)_PCL_ |
| 1723 | Stretching | PCL | C=O, crystalline^[35][36]^ | *v*(C=O) _PCL_ |
| 1705 | Stretching | PCL | Terminal O-H group of each polyester can exhibit hydrogen bonding interactions with C=O groups;^[37]^ C=O stretching in amorphous regions that are hydrogen bonded | *v*(C=O)_PCL_ |
| 1688 | Stretching | PCL | C=O, crystalline (H-bonded)^[37]^ | *v*(C=O)_PCL_ |
| 1479 | Bending | PCL | CH_2_, crystalline^[38]^ | δ(CH_2_)_PCL_ |
| 1470 | Bending | PCL | CH_2_, crystalline^[32][39][38]^ | δ(CH_2_)_PCL_ |
| 1454 | Asymmetric Bending | PLA | δ_as_(CH_3_)^[40]^ | δ_as_(CH_3_)PLA |
| 1419 | Wagging | PCL | CH_2_^[39]^ | γ(CH_2_)_PCL_ |
| 1382 | Symmetric Bending | PLA | CH_3_^[40]^ | δ_s_(CH_3_)_PLA_ |
| 1366 | Symmetric Bending | PLA | CH_3_^[40]^ | δ_s_(CH_3_)_PLA_ |
| 1361 | Bending | PCL | CH_2_^[41]^ | δ(CH_2_)_PCL_ |
| 1360 | Bending | PLA | CH^[40]^ | δ(CH)_PLA_ |
| 1318 |  | PLA | SC^[42][43]^ | 3_1_ helical chain conformation |
| 1306 |  | PLA | SC^[42]^ | 3_1_ helical chain conformation |
| 1267 | Asymmetric Stretching, Bending | PLA | -C-O-C, -CH^[44]^ | ν(C-O-C)_As,PLA_, δ(C–H),_PLA_ |
| 1240 | Asymmetric Stretching | PCL | C-O-C, crystalline^[38][45]^ | ν_as_(COC), PCL |
| 1210 | Asymmetric Stretching | PLA | C-O-C^[40]^ | ν_as_(COC), PLA |
| 1182 | Rocking | PLA | *r*_as_(CH_3_)^[40]^ | *r*_as_(CH_3_),PLA |
| 1128 | Rocking | PLA | r_as_(CH_3_)^[40]^ | r_as_(CH_3_),PLA |
| 1082 | Symmetric Stretching | PLA | C-O-C^[40]^ | ν_s_(COC), PLA |
| 1039 | Stretching | PLA | C–CH_3_^[40]^ | ν(C–CH_3_)_PLA_ |
| 1039 |  | PLA | -C-CH_3_, SC^[44]^ |  |
| 962 |  | PCL | -CH_2_–CH_2_–CH_2_–CH_2_–CH_2_– crystalline ordering^[38]^ | All-trans conformational ordering (*tttt*) |
| 956 | Rocking | PLA | CH_3_^[40]^ | r(CH_3_)_PLA_ |
| 934 |  | PCL | CH_2_-CH_2_-CH_2_-CH_2_-CH_2_- crystalline ordering^[38]^ | *tttt* ordering |
| 908 | - | PLA | 3_1_ helical chain conformation of SCs^[44][46]^ | 3_1_ helical chain conformation |
| 871 | Stretching | PLA | C–COO^[40]^ | ν(C–COO)_PLA_ |
| 841 | - | PCL | CH_2_–CH_2_–CH_2_–CH_2_–CH_2_– crystalline ordering^[38]^ | *tttt* ordering |

**v* is stretching, δ is bending, r is rocking, γ is wagging, *s* represents symmetric, *as* represents asymmetric.^[40]^

Note: There is no band present at 923 cm^-1^, which is characteristic of α crystal helices of PLA ν(C–COO).^[40]^

Based on the foundation from Noda et al., we use dynamic spectra (i.e., spectra obtained following application of an external perturbation) to develop 2D correlation spectra. Here, we use the increasing concentration of PCL in our aerogels as the external perturbation of these dynamic spectra, with the neat PLLA/PDLA control used as the reference spectrum for this 2D correlation analysis. As described by Noda et al., the synchronous 2D correlation intensity corresponds to the degree of similarity between two spectral band intensity variations, in function of the applied external perturbation.^[47]^ In contrast, the asynchronous 2D correlation intensity can be considered a measure of dissimilarity among structural variations (i.e., out-of-phase spectral variations).^[47]^

The synchronous 2D correlation intensity $\Phi$ at any given coordinate in the 2D correlation spectrum (i.e., at x-y coordinates corresponding to wavenumber values of *v*_1_ and *v*_2_, respectively) can be given by the relationship:^[47]^

$$\Phi(v_{1},v_{2})=\frac{1}{m-1}\sum_{j=1}^{m} \tilde{y}_{j}(v_{1})\cdot\tilde{y}_{j}(v_{2})$$

(S8)

Where $\tilde{y}_{j}(v_{i})$ is the FTIR spectral intensity value (i.e., transmittance) at a perturbation (i.e., PCL concentration) value of *j*, *m* is the number of dynamic spectra used in 2D correlation analysis. It should be noted that this analysis is more appropriately applied when perturbations are applied to the system in equally spaced increments. As such, we consider concentration perturbations of 5 and 10 wt.% PCL for 2D correlation analysis, with PLLA/PDLA control aerogels used as the reference spectrum. The synchronous 2D correlation spectrum represents simultaneous/coincidental changes in spectral intensity that occur when the perturbation is applied; this spectrum is symmetrical with respect to the diagonal (i.e., when *v*_1_ = *v*_2_).^[47]^ Here, peaks correlated with one another manifest at both diagonal and off-diagonal positions on the synchronous 2D correlation spectrum.^[47]^ Here, peaks along the diagonal (i.e., auto peaks) represent the susceptibility of the spectral intensity to change in function of the applied perturbation, and always present positive values. In contrast, cross peaks (i.e., peaks located off-diagonal) represent simultaneous/coincidental changes in spectral intensities at two different spectral variables (i.e., *v*_1_ and *v*_2_), implying possible correlations in the origin of these two spectral variations, within a correlation square drawn from corresponding auto peaks. Auto peaks that can be connected by cross-peaks (i.e., off-diagonal) within a correlation square are thus synchronously correlated. Although auto peaks are always positive, cross-peaks may be positive or negative, with positive cross-peaks indicating that the spectral intensity at cross-peak spectral coordinates *v*_1_ and *v*_2_ are increasing or decreasing together when the perturbation is applied. In contrast, negative cross peaks suggest that one of the spectral intensities is increasing, while the other decreases (i.e., concomitant changes in spectral band intensities, but opposite in direction as opposed to the spectral intensities moving in the same direction).

Furthermore, the asynchronous 2D correlation intensity spectrum is given by the following relationship:

$$\Psi\left( v_{1},v_{2} \right)=\frac{1}{m-1}\sum_{j=1}^{m} \tilde{y}_{j}(v_{1})\cdot\sum_{k=1}^{m} N_{jk}\cdot\tilde{y}_{k}(v_{2})$$

(S9)

Here, $N_{jk}$ corresponds to the *j*th row and *k*th column of the discrete Hilbert-Noda transformation matrix, described elsewhere.^[47]^ An asynchronous spectrum is antisymmetric with respect to the diagonal, and shows no auto peaks (i.e., only shows cross peaks off-diagonal). Analogous to synchronous spectra, correlation squares can be constructed for asynchronous spectra via the diagonal line. Asynchronous cross peaks appear if the spectral intensities of two spectral bands change out-of-phase with each other, with one band changing intensity more rapidly or slowly than the other during application of the external perturbation.^[47]^ In general, if the intensity change at *v*_1_ occurs before *v*_2_ during sequential perturbation application (i.e., with increasing PCL loading), the cross peak will be positive. If the intensity change at *v*_2_ occurs before *v*_1_ during sequential perturbation application, the cross peak will become negative. Exceptions to this are discussed elsewhere.^[47]^

The 2D Correlation analysis applied in this work was conducted using the 2D Correlation plugin on OriginPro®. Homo correlation analysis (i.e., FTIR data is correlated with FTIR data) was selected using the FTIR spectral data obtained for the PLLA/PDLA control aerogel as the reference spectrum, and the spectral data obtained for aerogels containing 5 and 10 wt.% PCL as the dynamic spectra, with the increasing PCL loading applied as the perturbation (i.e., perturbation variables of 0, 5 and 10 wt.% PCL). Pareto Scaling was applied as was recommended in fundamental work to minimize noise in the correlation spectra (default scaling factor of 0.5 in OriginPro® Plugin).^[48]^

The synchronous spectrum of scCO_2_-dried aerogels in the range of 1650 – 1850 cm^-1^ is illustrated in **Figure 4D**. Here, a single noticeable, yet broad auto peak is observable near (1745, 1745 cm^-1^) corresponding to *v*(C=O)_S,PLA_, with a moderate-intensity cross peak at (1723, 1745 cm^-1^). This positive correlation of the cross peak corroborates that the band near 1723 cm^-1^ corresponds to the crystalline *v*(C=O)_PCL_. The presence of a cross peak implies a simultaneous or coincidental spectral variation synchronously with increasing PCL loading confirming the identity of this spectral band as belonging to PCL. In contrast, the asynchronous correlation spectrum for scCO_2_-dried aerogels can be found in **Figure 4E**. This spectrum illustrates distinctly independent (i.e., out-of-phase; delayed or accelerated) interactions between the PLA and PCL. It should be noted that we expect that for an immiscible polymer mixture, there be minimal correlation spectra intensity changes observable in the asynchronous spectra, owing to the lack of interactions between the two materials.^[49]^ This figure illustrates a positive cross peak near (1723 cm^-1^, 1745 cm^-1^, shown in red), suggesting that the spectral intensity at *v*_1_ increases more rapidly than *v*_2_ with increasing PCL loading; these interactions between the *v*(C=O)_PCL_ and the *v*(C=O)_S,PLA_ could be ascribed to an interpenetrated configuration of PCL with PLA, or interfacial interactions near the interphase of the materials. It should be noted that this interaction spans a very broad range of *v*_1_ and *v*_2_ values, implying profound interactions between all *v*(C=O)_PCL_ and *v*(C=O)_PLA_ environments. This can be plausibly ascribed to the localization of the PCL phase at the interspherulitic region of the PLA SCs, with the PCL interpenetrated with the broad range of amorphous chain conformers in PLA (1750 - 1800 cm^-1^), that together form an interphase. As well, a negative cross peak appears near (1705 cm^-1^, 1723 cm^-1^), suggesting that the spectral intensity at *v*_2_ increases more rapidly than *v*_1_ with increasing PCL loading, and corresponds to interactions between amorphous C=O groups that are hydrogen bonded, and crystalline *v*(C=O)_PCL_, implying a gradient between these two chemical environments. The increase in PCL crystalline *v*(C=O)_PCL_ band intensity more rapidly than the amorphous band counterpart with increasing PCL loading is consistent with the saturation of the SC interspherulitic region with PCL, and subsequent phase separation of PCL into ordered crystalline domains. This supports our hypothesis of interpenetration of PCL and PLA at the interspherulitic region of the SCs, as was elucidated via thermogravimetric analysis with only a small portion of the aerogels degrading at higher temperatures than the PLLA/PDLA control aerogel, in addition to calorimetric analysis evidencing the evolution of a crystalline fraction in PCL with increasing PCL loading. While the compatibility between PLA and PCL has been highly debated in literature, it is generally believed that PLA and PCL are immiscible^[50,51]^ or partially miscible^[52,53]^ under most conventional melt-compounding conditions unless specific compatibilization strategies (e.g. *in situ* fibrillation) are applied.^[54]^ However, under solution blending processes, the phase behavior between the two materials is less understood; we believe this is attributed to the possibility of solvents overcoming the diffusion limitations that typically limit interpenetration of high *M_W_* polymer materials. Once homogeneously mixed, it appears that subsequent thermal treatments (e.g., TIPS processes, drying procedures) that induce crystallization effectively govern the degree of phase separation between aerogel constituents.

# **Synchrotron Small Angle X-ray Scattering Analysis – Supporting Information**

**Table S5:** Parameters obtained from T-S model fitting from ~0.06 – 0.135 Å^-1^.

| Sample Name | a | c_1_ | c_2_ | A | *R^2^* |
| --- | --- | --- | --- | --- | --- |
| 0 wt.% PCL | 3.007 × 10^1^ | -7.110 × 10^3^ | 8.165 × 10^5^ | 8.704 × 10^1^ | 0.99932 |
| 1 wt.% PCL | 7.447 × 10^2^ | -1.828 × 10^5^ | 2.145 × 10^7^ | 8.818 × 10^1^ | 0.99941 |
| 5 wt.% PCL | 1.378 × 10^5^ | -3.261 × 10^7^ | 3.878 × 10^9^ | 8.813 × 10^1^ | 0.99906 |
| 10 wt.% PCL | 4.259 × 10^0^ | -1.009 × 10^3^ | 1.437 × 10^5^ | 9.390 × 10^1^ | 0.99972 |
| 25 wt.% PCL | 1.975 × 10^3^ | -3.963 × 10^5^ | 6.750 × 10^7^ | 9.728 × 10^1^ | 0.99947 |

**Table S6:** Comparison of calculated T-S inter-cluster distance, radius of gyration, and maximum inter-cluster distance from T-S and P(r) fittings with pore width at *dV* Peak (from N_2_ adsorption-desorption pore-size distributions).

| Sample Name | T-S inter-cluster Distance (nm) | *R_g_* (nm) | *D_max_* (nm) | *Pore Width at dV Peak (nm)* |
| --- | --- | --- | --- | --- |
| 0 wt.% PCL | 8.70 | 7.36 | 22.1 | 7.80 |
| 1 wt.% PCL | 8.82 | 7.42 | 22.29 | 7.45 |
| 5 wt.% PCL | 8.81 | 7.45 | 22.21 | 7.45 |
| 10 wt.% PCL | 9.39 | 7.83 | 22.68 | 8.53 |
| 25 wt.% PCL | 9.73 | 9.81 | 26.99 | 8.53 |

# **Mechanical Property – Supporting Information**

**Figure S14:** Stress-strain behaviour of select aerogels subject to 1 mm×min^-1^ compressive loading, with inset image obtained during compressive testing to demonstrate its brittle character.

**Figure S15:** Ashby plot illustrating envelope density with respect to compressive modulus for a variety of biopolymer-based aerogels and foams.^[55–77]^

**Table S7:** Envelope density of aerogels before and after 100 compressive cycles at 20% strain, and change in density after cyclic compression testing, as well as compressive strength after 1^st^ Compressive Cycle at 20% Strain.

| PCL Content (wt.%) | Average Initial Envelope Density (g×cm^-3^) | Average Density after 100 Compressive Cycles at 20% Strain (g×cm^-3^) | Density Change after 100 Compressive Cycles at 20% Strain (%) | Compressive Strength (MPa) after 1^st^ Compressive Cycle at 20% Strain (MPa) |
| --- | --- | --- | --- | --- |
| 0 | 0.132 ± 0.006 | 0.147 ± 0.009 | 11.6 ± 1.1 | 0.035 ± 0.016 |
| 1 | 0.130 ± 0.003 | 0.148 ± 0.006 | 14.4 ± 0.7 | 0.035 ± 0.019 |
| 5 | 0.137 ± 0.002 | 0.151 ± 0.009 | 10.2 ± 0.9 | 0.030 ± 0.014 |
| 10 | 0.126 ± 0.003 | 0.141 ± 0.002 | 12.5 ± 0.3 | 0.025 ± 0.0075 |
| 25 | 0.121 ± 0.006 | 0.135 ± 0.002 | 10.8 ± 0.6 | 0.011 ± 0.005 |

# **Thermal Insulation and Thermal Management Assessment – Supporting Information**

**Figure S16:** Schematics illustrating the **A)** thermal insulation and **B)** thermal management Experimental Setups. Created in BioRender. Tuccitto, A. (2026) <https://BioRender.com/5ry9tmd>

**Figure S17:** Thermal Camera images corresponding to **A)** 0 wt.% PCL and **B)** 25 wt.% PCL aerogel samples placed on a hot plate set to 150 °C for a duration of 10 min, measuring their thermal insulation performance. Maximum and minimum temperatures of thermal camera images are 168 and 48 ^⚬^C, respectively.

**Figure S18:** Thermal camera images illustrating the front (illuminated) and back surface of **A)** 0 and **B)** 25 wt.% PCL aerogels under radiative heating, demonstrating their heat dissipation and thermal management capabilities.

# **Cassie-Baxter Model Predictions– Supporting Information**

The Cassie-Baxter model (CB) model was developed in previous literature^[78]^ and describes heterogeneous wetting of a droplet on a textured surface possessing valleys and troughs. In the CB-model, contact between the droplet and surface occurs at the surface texture peaks, with water unable to wet the valleys of the surface.^[79]^ The CB-model equation is given as:

$$\cos\theta_{C}=f_{s}\cdot cos \theta_{Y}+f_{a}\times cos \theta_{a}$$

(S10)

Where *θ_C_* is the contact angle predicted from the CB-model, *f_s_* is the fraction of the solid surface area in contact with the liquid (i.e., *f_s_* = *1 – Porosity*), *f_a_* is the fraction of surface occupied by air (i.e., Porosity), *θ_Y_* is the intrinsic contact angle for a smooth surface of the material, and *θ_a_* is the contact angle of air (assumed to be 180 °). It should be noted that the values for the PLA and PCL contact angles are 75.7 and 77.5 °, respectively.^[80][81][82]^ Rule of mixtures (i.e., *θ_Blend_* = *f_PLA_×θ_PLA_* + *f_PCL_×θ_PCL_*) was used for contact angle estimates of blends with negligible surface roughness. This yields the following CB-model used for calculation:^[83]^

$$\cos\theta_{C}=f_{s}\cdot cos \theta_{Y}+f_{a}\times(-1)$$

(S11)

As shown in **Table S8**, the CB-model WCA values align well (within error) with experimentally measured WCA values for all prepared aerogels.

# **Mechanistic Origin of Superhydrophobicity and Superoleophilicity**

Fundamentally, the experimentally observed combination of superhydrophobicity and superoleophilicity in the prepared aerogels can be ascribed to the pressure needed for a fluid to intrude a pore (i.e., intrusion pressure).^[84]^ Specifically, when the contact angle of the fluid > 90 º, the capillary pressure is positive, meaning that the aerogel can sustain a pressure difference across the pore, such that the fluid cannot intrude. In contrast, if the contact angle of the fluid < 90 º, the intrusion pressure is negative, with the fluid at the surface spontaneously absorbed without the need for applied pressure. Given the much lower surface tension of oils (e.g., hexane ~ 18.4 mN ·m^-1^) compared to water (~72 mN·m^-1^), as well as the hierarchically porous nature of the prepared aerogels, we believed that low surface tension fluids like hexanes can be readily absorbed by the aerogels owing to the negative intrusion pressure across the multi-length scale pores, while high surface tension fluids like water would still possess air entrapped between the pores that prevents fluid intrusion.

**Table S8:** Comparison between experimental and CB model estimates of water contact angles on the aerogels surface.

| Sample Name | Cassie-Baxter Model WCA (°) | Experimental WCA (°) |
| --- | --- | --- |
| 0 wt.% PCL | 150.1 | 149.5 ± 4.7 |
| 1 wt.% PCL | 150.4 | 154.9 ± 4.3 |
| 5 wt.% PCL | 149.5 | 150.2 ± 5.3 |
| 10 wt.% PCL | 150.7 | 149.2 ± 6.3 |
| 25 wt.% PCL | 151.1 | 150.7 ± 5.6 |

# **Static Sorption Capacity – Supporting Information**

**Figure S19:** Static sorption capacity corresponding to aerogels immersed in either hexane, canola oil, or diesel for 10 min.

# **Relationship between Flux and Crystalline Superstructure Size – Supporting Information**

**Figure S20:** Flux with respect to crystalline superstructure size, with linear fit and corresponding *R^2^* value from linear regression, for the **A)** 1^st^, **B)** 2^nd^, **C)** 3^rd^, **D)** 4^th^, **E)** 5^th^, **F)** 6^th^, **G)** 7^th^, **H)** 8^th^, **I)** 9^th^, and **J)** 10^th^ sorption-desorption cycles.

# **Comparison with State-of-the-Art**

**Table S9:** Comparison between the aerogels prepared in this study with other state-of-the-art polymer aerogels and relevant monolithic structures.

| Aerogels | *S_BET_ (m^2^ g^-1^)* | *T_m_* (°C) | ρ  (g cm^-1^) | *E_comp_* (MPa) | σ_comp_ (MPa)* | *k_Cond_*  (mW m^-1^ K^-1^) | WCA (°) | Q (g g^-1^)** | Flux (L m^-2^ hr^-1^)‡ | Recyclable? | Ref. |
| --- | --- | --- | --- | --- | --- | --- | --- | --- | --- | --- | --- |
| PLA Stereocomplex-based Aerogels | | | | | | | | | | | |
| *sc*PLA | 132 | 227 | 0.09 | 3.3 | ~ 0.15 | - | 149 | - | - | Yes | ^[85]^ |
| *sc*PLA | - | 218.9 | - | - | - | - | 140.1 | 24 | - | Yes | ^[86]^ |
| *sc*PLA | - | 215 | - | - | 0.1*** | 37 | 152 | - | - | Yes | ^[87]^ |
| scPLA/CNT | - | 220 | - | - | - | - | 159 | 20.8 | - | No | ^[88]^ |
| scPLA/GF | - | 215 | - | - | ~0.035 | 36.26 | 150 | - | - | No | ^[89]^ |
| State-of-the-art Aerogels and Hybrid Aerogel-Foams | | | | | | | | | | | |
| PU | 81 | - | 0.135 | 0.248 | - | 37 | - | - | - | No | ^[90]^ |
| Cellulose | 186 | - | ~ 0.06 | 5.84 | ~ 0.3 | 47.9 | - | - | - | - | ^[91]^ |
| PI/GO | - | - | ~ 0.05 | 25 | - | 35 | - | - | - | No | ^[92]^ |
| PI/silica | - | - | ~ 0.05 | 0.1 | ~ 0.005 | ~20 | - | - | - | No | ^[93]^ |
| PI/silica | - | - | - | 1.96 | ~ 0.28 | 31.1 | 125 | - | - | No | ^[94]^ |
| Melamine Foam | - | - | 0.010 | - | - | 35 | - | - | - | No | ^[95]^ |
| Melamine Foam-Silica | 409.4 | - | 0.060 | - | - | 51 | - | - | - | No | ^[95]^ |
| Melamine Foam-Silica | 471.9 | - | 0.037 | - | - | 42 | ~159 | - | - | No | ^[95]^ |
| Melamine Foam-Silica | 301.3 | - | 0.036 | - | - | 29 | ~152 | - | - | No | ^[95]^ |
| Melamine Foam-Silica | 62.8 | - | 0.014 | - | - | 27 | ~154 | - | - | No | ^[95]^ |
| Melamine Foam-Silica | 601.2 | - | 0.091 | - | - | 71 | - | - | - | No | ^[95]^ |
| Melamine Foam-Silica | 553.7 | - | 0.089 | - | - | 49 | ~156 | - | - | No | ^[95]^ |
| Melamine Foam-Silica | 349.8 | - | 0.039 | - | - | 33 | ~151 | - | - | No | ^[95]^ |
| Melamine Foam-Silica | 241.5 | - | 0.013 | - | - | 29 | ~153 | - | - | No | ^[95]^ |
| MOF /cellulose acetate foam | 35.3 | - | - | - | - | - | ~141 | ~7 | - | Yes | ^[84]^ |
| MOF /cellulose acetate foam | - | - | - | - | - | - | ~146 | ~8.4 | - | Yes | ^[84]^ |
| MOF /cellulose acetate foam | 52.1 | - | - | - | - | - | 153 | ~9 | 3250 | Yes | ^[84]^ |
| MOF /cellulose acetate foam | - | - | - | - | - | - | ~150 | ~8.6 | - | Yes | ^[84]^ |
| Bridged silica aerogels | - | - | - | - | - | - | ~140 | ~10 | 6872 | No | ^[96]^ |
| Aramid/PI | - | - | 0.005 | 0.004 | ~0.001 | 28.6 | - | - | - | No | ^[97]^ |
| PI | - | - | 0.089 | 1.59 | 0.3 | 27.5 | 0 | - | - | No | ^[98]^ |
| PI | - | - | 0.083 | 2.4 | ~0.3 | 32.5 | - | - | - | No | ^[99]^ |
| Thermoplastic Aerogels | | | | | | | | | | | |
| PLA | 85 | - | - | - | - | - | - | - | - | Yes | ^[100]^ |
| PEEK**** | 350 | - | ~ 0.19 | ~3 | - | - | 151.1 | - | - | Yes | ^[101]^ |
| *sc*PMMA | 357.9 | 193.7 | 0.03 | - | - | 23.9 | 141.6 | 29.8 | - | Yes | ^[102]^ |
| *sc*PMMA | 398.2 | 197.7 | 0.17 | 7.04 | 1.05 | 29.7 | 122.3 | 7.8 | - | Yes | ^[102]^ |
| *sc*PMMA | 367.8 | 195.3 | 0.40 | 79.3 | 8.74 | 65.1 | 103.7 | 2.9 | - | Yes | ^[102]^ |
| This Work | | | | | | | | | | | |
| *0 wt.% PCL* | **175** | **221.7** | **0.132** | **0.3130** | **0.0347** | **36.1** | **149.5** | **6.64** | **13224** | **Yes** | **-** |
| *1 wt.% PCL* | **186** | **220.8** | **0.130** | **0.2850** | **0.0351** | **37.6** | **154.9** | **6.59** | **13156** | **Yes** | **-** |
| *5 wt.% PCL* | **168** | **221.7** | **0.137** | **0.2688** | **0.0295** | **39.5** | **150.2** | **6.03** | **17352** | **Yes** | **-** |
| *10 wt.% PCL* | **110** | **223.2** | **0.126** | **0.2329** | **0.0253** | **36.9** | **149.2** | **6.56** | **5881** | **Yes** | **-** |
| *25 wt.% PCL* | **63** | **223.2** | **0.121** | **0.0861** | **0.0112** | **36.6** | **150.7** | **7.18** | **12009** | **Yes** | **-** |

*T_m_* = melting temperature, ρ = density, *E_comp_* = compressive modulus, σ_comp_ = compressive strength, *k_Cond_* = thermal conductivity. Tilde symbols are presented where values have been estimated based on data provided.

*Values at 20% strain.

**Data for a fluid with a density of ~0.95.

***The compressive strength was measured at 10% strain.

****The samples containing 10 wt.% of PEEK is considered.

‡Flux values are reported for hexanes. It should be noted that in literature, pumping systems differ among publications. As such, the applied vacuum pressure may differ. Nevertheless, these values are provided for the sake of comparison.

# **Demonstration of Material Re-processing**

**Figure S21:** Recycling or re-processing of the 0 wt.% PCL and 25 wt.% PCL aerogels via **A)** chemical recycling (aerogels were dissolved in DMF at 100 °C for 30 min). Images illustrating **B)** 0 wt.% and **C)** 25 wt.% PCL aerogel-derived films after melt re-processing (i.e., compression molding).

# **Statistical Analysis – Quantitative Evidence**

**Table S10:** Statistical analysis conducted using GraphPad Prism including One-way ANOVA and Dunnett’s Multiple Comparison Test.

|  |  | ANOVA | | Dunnett’s Multiple Comparison with 0 wt.% PCL (Control) | |
| --- | --- | --- | --- | --- | --- |
| Figure/ Table | **Data Description** | **F (Dfn,DFd)** | **ANOVA P Value** | **Sample Name** | **Adjusted p Value** |
| Fig. 6B | Compressive Modulus | F (4, 14) = 5.498 | P = 0.0071 |  |  |
|  | (n = 4) |  |  | 0 wt.% PCL | − |
|  | (n = 5) |  |  | 1 wt.% PCL | 0.9315 |
|  | (n = 4) |  |  | 5 wt.% PCL | 0.7810 |
|  | (n = 3) |  |  | 10 wt.% PCL | 0.3967 |
|  | (n = 3) |  |  | 25 wt.% PCL | 0.0026 |
| Table S7 | Compressive Strength | F (4, 14) = 1.589 | P = 0.2318 |  |  |
|  | (n = 4) |  |  | 0 wt.% PCL | − |
|  | (n = 5) |  |  | 1 wt.% PCL | − |
|  | (n = 4) |  |  | 5 wt.% PCL | − |
|  | (n = 3) |  |  | 10 wt.% PCL | − |
|  | (n = 3) |  |  | 25 wt.% PCL | − |
| Fig. 7A | Thermal Conductivity | F (4, 21) = 1.538 | P = 0.2276 |  |  |
|  | (n = 5) |  |  | 0 wt.% PCL | − |
|  | (n = 5) |  |  | 1 wt.% PCL | − |
|  | (n = 5) |  |  | 5 wt.% PCL | − |
|  | (n = 5) |  |  | 10 wt.% PCL | − |
|  | (n = 6) |  |  | 25 wt.% PCL | − |
| Table S1 | Porosity | F (4, 10) = 4.040 | P = 0.0334 |  |  |
|  | (n = 3) |  |  | 0 wt.% PCL | − |
|  | (n = 3) |  |  | 1 wt.% PCL | 0.9124 |
|  | (n = 3) |  |  | 5 wt.% PCL | 0.3218 |
|  | (n = 3) |  |  | 10 wt.% PCL | 0.4787 |
|  | (n = 3) |  |  | 25 wt.% PCL | 0.2071 |
| Fig. 8D | Water Contact Angle | F (4, 108) = 4.539 | P = 0.0020 |  |  |
|  | (n = 24) |  |  | 0 wt.% PCL | − |
|  | (n = 24) |  |  | 1 wt.% PCL | 0.0021 |
|  | (n = 20) |  |  | 5 wt.% PCL | 0.9771 |
|  | (n = 21) |  |  | 10 wt.% PCL | 0.9992 |
|  | (n = 24) |  |  | 25 wt.% PCL | 0.8602 |
| Fig. S19 | Sorption Capacity – Diesel | F (4, 11) = 6.674 | P = 0.0056 |  |  |
|  | (n = 4) |  |  | 0 wt.% PCL | − |
|  | (n = 3) |  |  | 1 wt.% PCL | 0.6266 |
|  | (n = 3) |  |  | 5 wt.% PCL | 0.9993 |
|  | (n = 3) |  |  | 10 wt.% PCL | 0.1054 |
|  | (n = 3) |  |  | 25 wt.% PCL | 0.0027 |
| Fig. S19 | Sorption Capacity – Canola Oil | F (4, 10) = 5.560 | P = 0.0128 |  |  |
|  | (n = 3) |  |  | 0 wt.% PCL | − |
|  | (n = 3) |  |  | 1 wt.% PCL | 0.9975 |
|  | (n = 3) |  |  | 5 wt.% PCL | 0.0913 |
|  | (n = 3) |  |  | 10 wt.% PCL | 0.9881 |
|  | (n = 3) |  |  | 25 wt.% PCL | 0.1557 |
| Fig. S19 | Sorption Capacity – Hexane | F (4, 12) = 2.810 | P = 0.0739 |  |  |
|  | (n = 3) |  |  | 0 wt.% PCL | − |
|  | (n = 3) |  |  | 1 wt.% PCL | − |
|  | (n = 4) |  |  | 5 wt.% PCL | − |
|  | (n = 4) |  |  | 10 wt.% PCL | − |
|  | (n = 3) |  |  | 25 wt.% PCL | − |
| Table S7 | Porosity after 20% Strain, 100 Cycles | F (4, 10) = 2.117 | P = 0.1532 |  |  |
|  | (n = 3) |  |  | 0 wt.% PCL | − |
|  | (n = 3) |  |  | 1 wt.% PCL | − |
|  | (n = 3) |  |  | 5 wt.% PCL | − |
|  | (n = 3) |  |  | 10 wt.% PCL | − |
|  | (n = 3) |  |  | 25 wt.% PCL | − |

# **References**

[1]	M. Fashandi, Z. Ben Rejeb, H. E. Naguib, C. B. Park, *Sep. Purif. Technol.* **2023**, *325*, 124201.

[2]	G. L. Loomis, J. R. Murdoch, K. H. Gardner, *Polym. Prepr. (Am. Chem. Soc. Div. Polym. Chem.)* **1990**, *31*, 55.

[3]	F. B. Khambatta, F. Warner, T. Russell, R. S. Stein, *J Polym Sci Part A-2 Polym Phys* **1976**, *14*, 1391.

[4]	M. Nagata, Y. Yamamoto, *J. Polym. Sci. A Polym. Chem.* **2009**, *47*, 2422.

[5]	TA Instruments, *Choosing Conditions in Modulated DSC*, **n.d.**

[6]	A. V Tuccitto, A. Anstey, N. D. Sansone, C. B. Park, P. C. Lee, *Int. J. Biol. Macromol.* **2022**, *218*, 22.

[7]	A. F. G. Leontowich, A. Gomez, B. D. Moreno, D. Muir, D. Spasyuk, G. King, J. W. Reid, C. Y. Kim, S. Kycia, *J. Synchrotron Radiat.* **2021**, *28*, 961.

[8]	B. H. Toby, R. B. Von Dreele, *Appl. Crystallogr.* **2013**, *46*, 544.

[9]	J. B. Hopkins, *J. Appl. Crystallogr.* **2024**, *57*, 194.

[10]	M. Embabi, M. S. Kweon, A. Anstey, A. V. Tuccitto, C. B. Park, M. E. Shivokhin, P. C. Lee, *ACS Appl. Polym. Mater.* **2023**, *5*, 2417.

[11]	X. Liu, M. Zhang, Y. Hou, Y. Pan, C. Liu, C. Shen, *Adv. Funct. Mater.* **2022**, *32*, 1.

[12]	N. D. Sansone, R. Aguiar, M. Embabi, N. Cheung, A. V. Tuccitto, N. R. S. Chang, M. Leroux, P. C. Lee, *Adv. Funct. Mater.* **2025**, *2420744*, 1.

[13]	R. Aguiar, N. D. Sansone, N. Cheung, A. V. Tuccitto, T. Y. T. Su, I. Soltani, M. Leroux, P. C. Lee, *Adv. Funct. Mater.* **2024**, *2403728*, DOI 10.1002/adfm.202403728.

[14]	R. Androsch, *Polymer (Guildf).* **1999**, *40*, 2805.

[15]	A. Toda, C. Tomita, M. Hikosaka, Y. Saruyama, *Polymer (Guildf).* **1998**, *39*, 5093.

[16]	A. Toda, C. Tomita, M. Hikosaka, Y. Saruyama, *Thermochim. Acta* **1998**, *324*, 95.

[17]	B. B. Sauer, W. G. Kampert, E. Neal Blanchard, S. A. Threefoot, B. S. Hsiao, *Polymer (Guildf).* **2000**, *41*, 1099.

[18]	J. Shao, S. Xiang, X. Bian, J. Sun, G. Li, X. Chen, *Ind. Eng. Chem. Res.* **2015**, *54*, 2246.

[19]	Y. F. Huang, Z. C. Zhang, Y. Li, J. Z. Xu, L. Xu, Z. Yan, G. J. Zhong, Z. M. Li, *Cryst. Growth Des.* **2018**, *18*, 1613.

[20]	W. W. Gerberich, W. Yu, D. Kramer, A. Strojny, D. Bahr, E. Lilleodden, J. Nelson, *J. Mater. Res.* **1998**, *13*, 421.

[21]	S. A. Epshtein, F. M. Borodich, S. J. Bull, *Appl. Phys. A Mater. Sci. Process.* **2015**, *119*, 325.

[22]	P. Opaprakasit, M. Opaprakasit, P. Tangboriboonrat, *Appl. Spectrosc.* **2007**, *61*, 1352.

[23]	Q. Ren, M. Wu, Z. Weng, X. Zhu, W. Li, P. Huang, L. Wang, W. Zheng, M. Ohshima, *Carbohydr. Polym.* **2022**, *276*, 118800.

[24]	J. Zhang, H. Sato, H. Tsuji, I. Noda, Y. Ozaki, *Macromolecules* **2005**, *38*, 1822.

[25]	Y. Xie, S. Y. H. Abdalkarim, Z. Ji, Z. Chen, M. Wang, H. Y. Yu, *ACS Sustain. Chem. Eng.* **2023**, *11*, 13164.

[26]	Z. Li, M. Zhang, X. Fan, X. Ye, Y. Zeng, H. Zhou, W. Guo, Y. Ma, J. Shao, C. Yan, *J. Polym. Sci. B Polym. Phys.* **2019**, *57*, 83.

[27]	P. Xie, J. Wang, J. Li, Q. Cheng, K. Zhou, J. Ren, *J. Polym. Sci. A Polym. Chem.* **2019**, *57*, 814.

[28]	A. Gupta, V. Katiyar, *ACS Sustain. Chem. Eng.* **2017**, *5*, 6835.

[29]	J. Liu, X. Qi, Q. Feng, Q. Lan, *Macromolecules* **2020**, *53*, 3493.

[30]	T. Elzein, M. Nasser-Eddine, C. Delaite, S. Bistac, P. Dumas, *J. Colloid Interface Sci.* **2004**, *273*, 381.

[31]	C. M. A. E. D. G. M. S. I.Noda, *AIP Conf. Proc.* **2000**, *503*, 77.

[32]	E. M. Abdelrazek, A. M. Hezma, A. El-khodary, A. M. Elzayat, *Egyptian Journal of Basic and Applied Sciences* **2016**, *3*, 10.

[33]	E. Meaurio, E. Zuza, N. López-Rodríguez, J. R. Sarasua, *Journal of Physical Chemistry B* **2006**, *110*, 5790.

[34]	E. Meaurio, N. López-Rodríguez, J. R. Sarasua, *Macromolecules* **2006**, *39*, 9291.

[35]	C. A. Kelly, K. L. Harrison, G. A. Leeke, M. J. Jenkins, *Polym. J.* **2013**, *45*, 188.

[36]	Y. He, Y. Inoue, *Polym. Int.* **2000**, *49*, 623.

[37]	C. Tian, S. Y. Fu, Q. J. Meng, L. A. Lucia, *Cellulose* **2016**, *23*, 2457.

[38]	Y. Duan, J. Zhang, H. Chang, S. Yan, C. Yang, I. Takahashi, Y. Ozaki, *Macromolecules* **2010**, *43*, 5315.

[39]	K. Phillipson, J. N. Hay, M. J. Jenkins, *Thermochim. Acta* **2014**, *595*, 74.

[40]	P. Pan, J. Yang, G. Shan, Y. Bao, Z. Weng, A. Cao, K. Yazawa, Y. Inoue, *Macromolecules* **2012**, *45*, 189.

[41]	D. Milovac, G. Gallego Ferrer, M. Ivankovic, H. Ivankovic, *Materials Science and Engineering C* **2014**, *34*, 437.

[42]	N. M. Praveena, P. Shaiju, R. B. A. Raj, E. B. Gowd, *Polymer (Guildf).* **2022**, *240*, 124495.

[43]	W. Li, J. Cao, L. Fu, F. Liu, Y. Huang, Y. He, L. Jiang, Y. Dan, *Int. J. Biol. Macromol.* **2024**, *261*, 129834.

[44]	W. Li, X. Wu, X. Chen, Z. Fan, *Eur. Polym. J.* **2017**, *89*, 241.

[45]	A. Elzubair, C. N. Elias, J. C. M. Suarez, H. P. Lopes, M. V. B. Vieira, *J. Dent.* **2006**, *34*, 784.

[46]	A. Michalski, M. Socka, M. Brzeziński, T. Biela, *Macromol. Chem. Phys.* **2018**, *219*, 4.

[47]	and Y. O. I. Noda, A. E. Dowrey, C. Marcott, G. M. Story, *Appl. Spectrosc.* **2000**, *54*, 230A.

[48]	I. Noda, *J. Mol. Struct.* **2008**, *883–884*, 216.

[49]	H. Huang, S. Malkov, M. Coleman, P. Painter, *Macromolecules* **2003**, *36*, 8156.

[50]	R. Dell’Erba, G. Groeninckx, G. Maglio, M. Malinconico, A. Migliozzi, *Polymer (Guildf).* **2001**, *42*, 7831.

[51]	P. F. M. Finotti, L. C. Costa, M. A. Chinelatto, *Macromol. Symp.* **2016**, *368*, 24.

[52]	J. T. Yeh, C. J. Wu, C. H. Tsou, W. L. Chai, J. D. Chow, C. Y. Huang, K. N. Chen, C. S. Wu, *Polymer - Plastics Technology and Engineering* **2009**, *48*, 571.

[53]	V. Mittal, T. Akhtar, N. Matsko, *Macromol. Mater. Eng.* **2015**, *300*, 423.

[54]	A. R. Kakroodi, Y. Kazemi, D. Rodrigue, C. B. Park, *Chemical Engineering Journal* **2018**, *351*, 976.

[55]	S. Takeshita, S. Yoda, *Chemistry of Materials* **2015**, *27*, 7569.

[56]	L. Wang, D. A. Schiraldi, M. Sánchez-Soto, *Ind. Eng. Chem. Res.* **2014**, *53*, 7680.

[57]	H. B. Chen, B. Sen Chiou, Y. Z. Wang, D. A. Schiraldi, *ACS Appl. Mater. Interfaces* **2013**, *5*, 1715.

[58]	S. Zhao, W. J. Malfait, E. Jeong, B. Fischer, Y. Zhang, H. Xu, E. Angelica, W. M. Risen, J. W. Suggs, M. M. Koebel, *ACS Sustain. Chem. Eng.* **2016**, *4*, 5674.

[59]	S. Zhao, W. J. Malfait, A. Demilecamps, Y. Zhang, S. Brunner, L. Huber, P. Tingaut, A. Rigacci, T. Budtova, M. M. Koebel, *Angewandte Chemie* **2015**, *127*, 14490.

[60]	P. Campia, E. Ponzini, B. Rossi, S. Farris, T. Silvetti, L. Merlini, M. Brasca, R. Grandori, Y. M. Galante, *Carbohydr. Polym.* **2017**, *158*, 102.

[61]	M. Ahmadi, A. Madadlou, A. A. Saboury, *Food Chem.* **2016**, *196*, 1016.

[62]	K. Finlay, M. D. Gawryla, D. A. Schiraldi, *Ind. Eng. Chem. Res.* **2008**, *47*, 615.

[63]	A. E. Donius, A. Liu, L. A. Berglund, U. G. K. Wegst, *J. Mech. Behav. Biomed. Mater.* **2014**, *37*, 88.

[64]	S. F. Plappert, J. M. Nedelec, H. Rennhofer, H. C. Lichtenegger, F. W. Liebner, *Chemistry of Materials* **2017**, *29*, 6630.

[65]	M. Schestakow, I. Karadagli, L. Ratke, *Carbohydr. Polym.* **2016**, *137*, 642.

[66]	B. Wicklein, A. Kocjan, G. Salazar-Alvarez, F. Carosio, G. Camino, M. Antonietti, L. Bergström, *Nat. Nanotechnol.* **2015**, *10*, 277.

[67]	J. Fu, S. Wang, C. He, Z. Lu, J. Huang, Z. Chen, *Carbohydr. Polym.* **2016**, *147*, 89.

[68]	Y. Kobayashi, T. Saito, A. Isogai, *Angewandte Chemie* **2014**, *126*, 10562.

[69]	M. Pääkkö, J. Vapaavuori, R. Silvennoinen, H. Kosonen, M. Ankerfors, T. Lindström, L. A. Berglund, O. Ikkala, *Soft Matter* **2008**, *4*, 2492.

[70]	H. Qi, E. Mäder, J. Liu, *J. Mater. Chem. A Mater.* **2013**, *1*, 9714.

[71]	H. Sehaqui, Q. Zhou, L. A. Berglund, *Compos. Sci. Technol.* **2011**, *71*, 1593.

[72]	H. Sehaqui, M. Salajková, Q. Zhou, L. A. Berglund, *Soft Matter* **2010**, *6*, 1824.

[73]	H. Wang, Z. Shao, M. Bacher, F. Liebner, T. Rosenau, *Cellulose* **2013**, *20*, 3007.

[74]	S. Zhao, Z. Zhang, G. Sèbe, R. Wu, R. V. Rivera Virtudazo, P. Tingaut, M. M. Koebel, *Adv. Funct. Mater.* **2015**, *25*, 2326.

[75]	T. Zheng, A. Li, Z. Li, W. Hu, L. Shao, L. Lu, Y. Cao, Y. Chen, *RSC Adv.* **2017**, *7*, 34461.

[76]	F. Fischer, A. Rigacci, R. Pirard, S. Berthon-Fabry, P. Achard, *Polymer (Guildf).* **2006**, *47*, 7636.

[77]	I. Karadagli, B. Milow, L. Ratke, B. Schulz, G. Seide, T. Gries, *Synthesis and Characterization of Highly Porous Cellulose Aerogels for Textiles Applications*, **2012**.

[78]	A. B. D. Cassie, S. Baxter, **1944**, 546.

[79]	Y. Gu, W. Zhang, J. Mou, S. Zheng, L. Jiang, Z. Sun, E. Wang, *Advances in Mechanical Engineering* **2017**, *9*, 1.

[80]	U. Saeed, A. A. Taimoor, S. U. Rathur, H. Alhumade, H. AlTuraif, H. S. Bamufleh, E. Siddiqui, B. Al-Zaitone, *J. Compos. Mater.* **2023**, *57*, 1363.

[81]	G. Damonte, B. Barsanti, A. Pellis, G. M. Guebitz, O. Monticelli, *Eur. Polym. J.* **2022**, *176*, 111402.

[82]	I. Sousa, A. Mendes, R. F. Pereira, P. J. Bártolo, *Mater. Lett.* **2014**, *134*, 263.

[83]	A. J. B. Milne, A. Amirfazli, *Adv. Colloid Interface Sci.* **2012**, *170*, 48.

[84]	S. Wang, Z. Gao, X. Qi, C. Li, Y. Xie, X. Yang, W. Song, Z. Liu, *J. Environ. Chem. Eng.* **2022**, *10*, DOI 10.1016/j.jece.2022.108521.

[85]	V. G. Krishnan, N. M. Praveena, R. B. A. Raj, K. Mohan, E. B. Gowd, *ACS Appl. Polym. Mater.* **2023**, *5*, 1556.

[86]	P. Chen, D. Bai, H. Tang, H. Liu, J. Wang, G. Gao, L. Li, *Polymer (Guildf).* **2022**, *255*, 2.

[87]	X. Liu, M. Zhang, Y. Hou, Y. Pan, C. Liu, C. Shen, *Adv. Funct. Mater.* **2022**, *32*, 1.

[88]	W. Zhang, M. Zhang, Q. Chen, X. Liu, *Int. J. Biol. Macromol.* **2024**, *263*, 130283.

[89]	S. Liao, D. Bai, Y. Jia, J. Sun, H. Liu, L. Li, M. Xu, *Int. J. Biol. Macromol.* **2024**, *274*, DOI 10.1016/j.ijbiomac.2024.133470.

[90]	A. Bang, C. Buback, C. Sotiriou-Leventis, N. Leventis, *Chemistry of Materials* **2014**, *26*, 6979.

[91]	I. Karadagli, B. Schulz, M. Schestakow, B. Milow, T. Gries, L. Ratke, *Journal of Supercritical Fluids* **2015**, *106*, 105.

[92]	T. Xue, W. Fan, X. Zhang, X. Zhao, F. Yang, T. Liu, *Compos. B Eng.* **2021**, *219*, DOI 10.1016/j.compositesb.2021.108963.

[93]	J. Tian, Y. Yang, T. Xue, G. Chao, W. Fan, T. Liu, *J. Mater. Sci. Technol.* **2022**, *105*, 194.

[94]	X. Zhang, X. Ni, C. Li, B. You, G. Sun, *J. Mater. Chem. A Mater.* **2020**, *8*, 9701.

[95]	M. Fashandi, Z. Ben Rejeb, H. E. Naguib, C. B. Park, *Sep. Purif. Technol.* **2023**, *325*, 124201.

[96]	Z. Ben Rejeb, A. Abidli, A. Zaoui, M. Fashandi, A. Selka, H. E. Naguib, C. B. Park, *One-Pot Synthesis of Rationally-Designed Flexible, Robust, and Hydrophobic Ambient-Dried Molecularly-Bridged Silica Aerogels with Efficient and Versatile Oil/Water Separation Applications*, Springer International Publishing, **2024**.

[97]	X. Zhang, X. Ni, M. He, Y. Gao, C. Li, X. Mo, G. Sun, B. You, *Mater. Chem. Front.* **2021**, *5*, 804.

[98]	O. A. Tafreshi, S. Ghaffari-Mosanenzadeh, S. Karamikamkar, Z. Saadatnia, S. Kiddell, C. B. Park, H. E. Naguib, *J. Mater. Chem. C Mater.* **2022**, *10*, 5088.

[99]	S. G. Mosanenzadeh, Z. Saadatnia, F. Shi, C. B. Park, H. E. Naguib, *Polymer (Guildf).* **2019**, *176*, 213.

[100]	A. Bueno, C. Luebbert, S. Enders, G. Sadowski, I. Smirnova, *J. Mater. Sci.* **2021**, *56*, 18926.

[101]	S. J. Talley, C. L. AndersonSchoepe, C. J. Berger, K. A. Leary, S. A. Snyder, R. B. Moore, *Polymer (Guildf).* **2017**, *126*, 437.

[102]	S. S. Rahman, A. V. Tuccitto, Z. Ben Rejeb, P. C. Lee, C. B. Park, *Small* **2025**, DOI 10.1002/smll.202508286.
